# The CATALYTIC tool to assess feasibility of implementing evidence-based interventions for cardiovascular diseases in 46 low- and middle-income countries: survey outcomes and tool reliability testing

**DOI:** 10.3389/fpubh.2025.1597996

**Published:** 2025-12-10

**Authors:** Temitope Ojo, Hanan Yassin, Esther Sowunmi, Tania Hameed, Nessa Ryan, Joyce Gyamfi, Donna Shelley, Olugbenga Ogedegbe, Emmanuel Peprah

**Affiliations:** 1 Division of Infectious Diseases, School of Medicine at Washington University in St Louis, St. Louis, MO, United States; 2 Department of Social and Behavioral Sciences, New York University School of Global Public Health, New York, NY, United States; 3 Department of Global and Environmental Health, New York University School of Global Public Health, New York, NY, United States; 4 Department of Public Health Policy and Management, New York University School of Global Public Health, New York, NY, United States; 5 Department of Population Health, NYU Langone Health-NYU Grossman School of Medicine, New York, NY, United States; 6 Institute for Excellence in Health Equity, NYU Langone Health, New York, NY, United States

**Keywords:** implementation feasibility, low- and middle-income countries, cardiovascular disease, context, tool development, CATALYTIC tool, measurement and evaluation

## Abstract

**Background:**

Evidence-based interventions (EBI) for cardiovascular disease (CVD) in low- and middle-income countries (LMIC) may face feasibility challenges due to the inadequacy of existing instruments. To address this, researchers developed the Contextual Index of Feasibility on Early-Stage Implementation in LMIC (CATALYTIC) tool, which integrates contextual factors into the assessment of feasibility.

**Methods:**

The tool’s items were developed through a systematic review and key informant interviews, and were later assessed for relevance and importance by 13 LMIC researchers and implementers employing a Delphi technique. The survey was then tested for usability by five individuals with CVD experience in LMIC. The CATALYTIC tool consists of 17 items that measure contextual factors that directly influence early-stage LMIC implementation. Descriptive analysis, logistic regression, item reliability using Cronbach’s alpha, and exploratory factor analysis (EFA) were performed on survey data.

**Results:**

In a survey of 216 respondents from 46 countries, 63.4 to 81.5% of respondents noted a significant impact of contextual factors on implementation feasibility, with high reliability (Cronbach’s alpha 0.88) for 12 items. The majority of interventions focused on patient-level care in rural settings. The survey items align primarily with constructs related to implementation climate and readiness for implementation, as well as inductive themes addressing existing needs and barriers to inform intervention design. The survey found diversity in geographic and experiential backgrounds, with significant representation from South Africa, Mexico, and India. Over a third identified as researchers, and 15% held multiple roles. The survey identified three distinct factors influencing how researchers and implementers assess CVD intervention feasibility in LMIC. A 6% increase in odds for moderately feasible interventions was linked to each point increase in the composite score of perceived contextual influence.

**Conclusion:**

Overall, the CATALYTIC tool with 12 reliable survey items can help researchers and implementers elucidate perceptions of contextual factors influencing the feasibility of CVD-related EBI in LMIC. The survey items reflect respondents’ practical focus in resource-limited settings and can inform intervention design by addressing existing needs and barriers. The tool’s integration of contextual factors into the assessment of feasibility can help overcome the inadequacy of existing instruments by providing more tailored and conceptually clear assessments of feasibility.

## Background

In low- and middle-income countries (LMIC), where over 75% of the global burden of deaths is due to cardiovascular disease (CVD) (1), there remains a knowledge and evaluation gap in the contextual feasibility of evidence-based CVD interventions (2). Assessing feasibility helps researchers and implementers to prioritize resources and manpower to achieve better health outcomes from their evidence-based interventions (EBI) through an efficient process. In LMIC, some factors (for example, limited resources, current health system set-ups, existing health policies and national guidelines for chronic care, national readiness/prioritization for healthcare reform or change, health insurance systems or health financing landscape, healthcare services utilization, and readiness of stakeholders and gatekeepers to engage in change processes/systems) have pronounced effects on the feasibility of implementing or bringing EBI to scale (3, 4).

As LMIC tackle the growing burden of CVD, it is essential that high-impact interventions addressing CVD and risk factors can be feasibly implemented in these settings (5). Inadequate assessment of feasibility contributes to suboptimal implementation of EBI and subsequently undesired intervention outcomes. Feasible implementation of high-impact CVD solutions such as controlling hypertension and reducing sodium and fatty acid intake in LMIC is projected to delay 84.7 million deaths by 2040 (6). Without assessing feasibility when implementing interventions in LMIC to address the CVD treatment gap, countries with fragile and overtaxed healthcare infrastructures are projected to spend unnecessary amounts of money, calculated at approximately $84 billion per decade, without significant improvements in health outcomes for CVD (7). Evidence-based and high-impact CVD interventions, which work in several high-income settings, might not necessarily achieve optimal positive outcomes when replicated in LMIC settings (2, 6).

According to the definition of Proctor et al. (8), feasibility is defined as “the extent to which an intervention is plausible in each agency or setting.” Despite evidence that supports the benefits and feasibility of high-impact interventions to address the most prevalent CVD risk factors, known as “best buys” (6), LMIC still underachieve optimal benefits of “best buys” (2). These high-impact interventions include scaling up the treatment of hypertension to 70%, reducing sodium intake by 30%, and eliminating the intake of artificial trans fatty acids (6). Persistent implementation barriers of CVD interventions in LMIC include but are not limited to poor awareness of CVD and its risk factors among vulnerable populations, programmatic shortfalls to improve cost-effectiveness, measurement and diagnosis challenges, suboptimal availability of trained healthcare personnel, and struggling health system capacity to adopt and sustain interventions (2). Given these LMIC-specific challenges in CVD prevention and management, the shortage of context-specific tools that can facilitate a rigorous assessment of how feasible it is to implement CVD EBIs, including ‘best buys,’ contributes to the underachievement of implementation efforts and health benefits of these interventions in LMIC.

To identify robust instruments assessing implementation outcomes as proposed by Proctor et al. (8), Lewis and colleagues assessed the psychometric properties of 104 instruments that measured the eight implementation outcomes, including feasibility (9). Of these instruments, only eight were identified to assess feasibility (10–17), but none of these feasibility instruments had an adequate score as defined by the authors on all six psychometric qualities—internal consistency, structural validity, predictive validity, norms, responsiveness, and usability (9, 18).

Feasibility is an essential yet underdeveloped implementation outcome, thus evaluating feasibility allows researchers and other implementers to identify early on the uncertainty that may hinder the scale-up of EBI (19, 20). Identifying uncertainty can allow researchers and policymakers to devote adequate resources to crystallize processes, build capacity, or to reallocate resources from unfeasible schemes, thereby saving time, money, and labor (5, 21). An accurate estimation of contextual factors that influence the feasibility of CVD interventions in LMIC can provide researchers and other implementation stakeholders with valuable information to equip their implementation plans and processes with the requisite quality and mix of conditions that guarantees optimal implementation feasibility and success, against the backdrop of systemic resource constraints, as is the reality in many LMIC settings (22, 23). A robust estimation of contextual influence on implementation feasibility will enable a systematic definition of feasibility parameters for implementing EBI in LMIC.

Despite the importance of feasibility as a fundamental implementation research outcome, there is a dearth of data on rigorous assessment of this outcome in existing studies (24–26). A scoping review showed that although CVD care interventions in LMIC exist and are presumed feasible (26), there was an inconsistency in the evaluation of feasibility across various research studies (26). This gap in adequate instrumentation propagates inconsistent methodology and reporting, thereby slowing the process of usability and translation of EBI to LMIC (27).

Given the importance of evaluating the feasibility of EBI in LMIC, it is essential to develop reliable psychometric tools for assessing feasibility at different stages of an intervention. For this study, we developed and tested a survey to elucidate the perceptions of individuals who implement CVD-related EBI about contextual factors that influence the feasibility of their interventions in LMIC settings. We used the two contextual domains of the Consolidated Framework for Implementation Research (CFIR)—inner setting and outer setting, to guide a systematic review and key informant interviews to generate survey items that captured the influence of contextual factors on implementation feasibility (4, 28). Using the Delphi method, a purposive sample of experts, researchers, and implementers of LMIC-based CVD interventions prioritized survey items by rating the items’ relevance and importance in capturing the extent of influence contextual factors have on implementing CVD interventions in LMIC. This survey was designed to capture quantifiable data to estimate the perceived extent of influence contextual factors have on implementation feasibility in LMIC. Also, the survey data was used to measure the reliability of the items to estimate the influence of contextual factors on implementation feasibility and psychometrically identify different dimensions (factors) to the concept of contextual influence on implementation feasibility.

## Methods

Informed by a systematic review, key informant interviews, and a Delphi process, we developed the **C**ontextual Index of Fe**A**sibili**T**y on E**A**r**LY**-Stage Implemen**T**ation in LM**IC** (CATALYTIC) tool, using a sequential exploratory mixed-methods design to capture the perceptions of researchers and implementers about contextual factors that influence the feasibility of implementing CVD-related EBI in LMIC settings (28). For this study, CVD-related EBI included a list of WHO guideline-recommendations of effective interventions, ranging from primary interventions that are early preventive strategies such as education and lifestyle counseling, behavior modifications, routine hypertension screening, to secondary interventions such as administering medications, to tertiary interventions, such as stroke rehabilitation or speech therapy (1). We collected one-time, cross-sectional data, which required recall in some instances (28). The qualitative component comprised of a systematic review and key informant interviews with nine participants. The quantitative portion consisted of a 2-round Delphi process with 13 participants and a survey tool.

### Phases of survey development

We developed the survey in three phases: item generation, item rating, and survey building with usability testing. We used data from a systematic review and key informant interviews to generate survey items. We utilized the Delphi process to gain consensus on survey items by rating each item on its relevance to and importance in assessing contextual influence. We built a survey tool on the Qualtrics platform and conducted retrospective cognitive testing of the items for user-friendliness and cognitive alignment (see Table 1).

**Table 1:** Analysis plan by survey development phases.

Phases	Methods	Analysis
Item generation	Systematic review	Nine reviewers screened and extracted relevant data from selected papers to identify factors related to CFIR inner setting and outer setting constructs, using these criteria: Studies were conducted in LMIC Reports on the feasibility of cardiovascular health interventions Reports on CFIR inner setting and outer setting constructs related to cardiovascular health interventions * CFIR contextual constructs that did not appear in the review were further investigated in the key informant interviews Qualitative content analysis was used to identify themes and assign contextual facilitators and barriers to CFIR inner-setting and outer-setting constructs (see Supplementary material 2)
	Key informant interview	Transcripts were analyzed using a deductive-inductive approach: Deductive coding based on *a priori* themes of feasibility and CFIR contextual constructs Inductive coding for themes not covered by deductive codes *Used qualitative analysis software NVivo (1.6.1) to organize codes and themes Identified and described contextual themes with illuminating quotes from key informants [28]. (see Supplementary material 3) Used review and interview themes identified from deductive CFIR inner setting and outer setting codes and inductive codes to create survey items Refined survey items 3 times with research team for grammatical, logical, and cognitive coherence
Item rating	Delphi	Assessed content validity of the survey items by estimating agreement across raters, on the extent to which the survey items were relevant and representative of the construct of contextual influence on feasibility [29]. Based on these ratings, items were ranked in the following order of consensus [29-31]: Positively rated as relevant and important Positively rated on one quality only Negatively rated on both qualities Selected items were rated as both relevant and important for the survey Shared expert feedback on items rated positively for one quality with raters and readministered rating questionnaire with only these items Items rated positively for both qualities in the 2^nd^ round were selected for the final survey; removed other items that did not meet the inclusion criteria Analyzed the frequency of raters’ agreement on the survey items using Microsoft Excel
Item pretesting	Retrospective think-aloud cognitive testing	Revised survey questions and prompts twice, incorporating feedback from 5 pretesters Transitioned survey to final online version using NYU Qualtrics platform. Administered survey to pretesters to confirm its user-friendliness and ease of completion in the online format.

#### Item generation

We used deductive and inductive methods to generate survey items from systematic review data and key informant interview transcripts. The systematic review of literature evidence included commonly used definitions of feasibility in implementation science, similar taxonomy and synonyms for feasibility, and commonly reported factors that influence the feasibility of CVD-related EBI. Major databases such as PubMed, Google Scholar were searched for qualifying studies. Nine key informants were interviewed over ZOOM to provide experiential depth to the contextual factors identified from the systematic review and to explore the experts’ perceptions about the concept of feasibility. They occupied roles of principal investigators and co-investigators on CVD research teams, with years of experience in LMIC-context CVD research ranging from 3 to 21 years. The average length of the interviews was 35 min.

Data from the systematic review and key informant interviews were analyzed qualitatively, and 66 survey items were created from the themes that emerged from the data (see Table 1). Four research personnel reviewed and revised the survey items before the item rating phase.

#### Item rating

For this study, the Delphi process was used to engage a group of experts anonymously to arrive at the most reliable consensus on the survey items that should be used to assess implementation feasibility in LMIC (29). A group of 13 experts, including implementation scientists and researchers, collaborated on a 2-round Delphi process (29–31) to agree on survey items that would best assess the feasibility of implementing health-related EBI in LMIC. Experts with experience implementing CVD interventions in LMIC rated the relevance and importance of each survey item. The two rounds of the Delphi rating was administered as a Qualtrics-based questionnaire, wherein the experts were asked to provide an explanation for disqualifying any item. For the first round, the initial list of 66 survey items were included in the questionnaire wherein participants rated each item on a binary response scale: (i) relevance in assessing feasibility in LMIC (“Relevant”/“Not Relevant”), and (ii) importance in evaluating feasibility (“Important”/“Not Important”). The second round was initiated to finalize agreement from all raters on survey items that were not decisively rated as relevant and important in the first round (see Table 1).

#### Survey building and pretesting

We created a survey from the final list of rated items, which we pretested with five professionals with varying degrees of expertise in implementation science or CVD-related research using retrospective think-aloud cognitive tests (32). Research personnel virtually interviewed pretesters about the survey items, the response scale, and the pretesters’ understanding of the items. The personnel also observed the pretesters while they completed the survey. The survey was revised at least twice based on feedback from pretesting and was transitioned into an online survey on New York University (NYU) Qualtrics. The survey was pretested again with all five professionals to ensure appropriate survey logic and flow in its final online format. Further feedback was used to revise the final online survey version that was administered to a larger sample of the target participants (see Table 1).

### Sample selection

The survey aimed to gather data on CVD related research and EBI in LMIC. The targeted participants were researchers, health providers, policymakers, healthcare administrators, and other implementers who have been part of implementing CVD-related research in LMIC and have piloted or are piloting CVD-related EBI in LMIC. To ensure a representative sample, the researchers used a purposive sampling approach and identified eligible participants from various professional and community organizations such as the National Institutes of Health John E. Fogarty International Center, Pan-African Society of Cardiologists (PASCAR), Global Alliance for Chronic Disease (GACD), Global Research on Implementation and Translation Science Consortium (GRIT), LinkedIn, Prolific, Global Digital Health Researchers Network, World Federation affiliates, LINKS, AFRO-NETS, and E-Drugs. The eligible participants for this survey were those who served in one or more of the following roles: researchers (33), implementers (34), health policymakers or planners (35), health administrators or managers (36), and health practitioners who deliver healthcare services either directly or indirectly (37). The researchers employed a non-random sampling approach to ensure that all categories and geographical regions of LMIC registered in any research and professional networks were represented in the sample (38). Primary contacts on different research projects were identified and asked to assist with sharing the survey with personnel whose roles fit the described roles of eligible professionals for this survey. The sampling approach was necessary as earlier literature reviews had shown a scarcity of reported CVD-related interventions and trained researchers and practitioners in LMIC. The goal was to ensure that the survey reached all eligible professionals to gather comprehensive data on CVD-related research and EBI in LMIC. A list of member countries in NIH-Fogarty, GRIT, GACD, and PASCAR can be found in Supplementary material 1.

### Data collection

The survey was self-administered once by participants via NYU Qualtrics, an online survey platform. Emails and social media posts on LinkedIn, X (formerly Twitter), and Facebook containing the survey link were sent to individuals at research and professional groups who may know eligible survey participants. Data collection started in March 2021 and was concluded in December 2021.

The average length of the survey was 15 min and was translated from English to Spanish, French, and Portuguese, all languages suitable for our target population. The 28-item survey contained 17 items, generated from the systematic review and key informant interviews, and 11 items on demography and research experiences of respondents. Responses were provided on a 5-point Likert scale, with 1 being the lowest quantity and 5 being the highest quantity of the measure captured. Fifteen of the 17 items were CFIR-coded survey items that captured the perceived extent of influence contextual factors have on the feasibility of CVD interventions, while two items were inductively coded. Other survey questions captured:

- Respondents’ agreement on the definition of feasibility (8)
- Respondents’ self-reports on the feasibility of their CVD interventions
- Characteristics of respondents’ LMIC-based research or implementation experiences and the nature of CVD interventions they implemented, such as years of experience, location, research population, and type of CVD research.

Data was analyzed for proportions (with percentages) of respondents’ answers to discrete, count, or categorized values, and the mean value with standard deviations (SD) for continuous variables like duration of interventions and years of experiences. The reliability of the survey items was estimated as Cronbach’s alpha, with average inter-item correlation coefficients (39, 40). Factor identification was done using exploratory factor analysis (EFA) (40, 41).

## Analysis

The statistical software Stata SE 17 was used to analyze the survey data. Descriptive analyses were conducted to measure the distribution of participants’ responses to survey questions. Summary statistics of demographic data included means and SDs, and proportions in percentages. The five-point Likert scale responses to the 17 survey items on contextual factors that influence feasibility, along with survey items that captured agreement on the definition of feasibility and self-reported ratings on intervention feasibility, were recoded into binary variables and analyzed as proportions.

Bivariate analysis was conducted using a logistic regression model to test for statistically significant associations (odds ratio [OR]); with a *p*-value of <0.05 and 95% confidence interval [CI]), which show that contextual factors directly influence how researchers or implementers rate the feasibility of CVD-related EBI in LMIC. We created composite variables derived by summing the respondents’ scores for 12 of the 17 survey items that asked about the extent of influence of contextual factors on implementing CVD interventions in LMIC. Similarly, we created composite variables for any factor derived from the EFA. The composite variables were the independent (continuous) variables in the regression model. We also recoded the self-reported responses on the feasibility of respondents’ most recent CVD interventions in LMIC into a binary variable, where responses originally coded as 1 (Not at all feasible) and 2 (A little feasible) were recoded as 0 (Low feasibility) and responses originally coded as 3 (Moderately feasible), 4 (Very feasible), and 5 (Extremely feasible) were recoded as 1 (Moderate-High feasibility). This binary variable was used as the dependent variable in the logistic regression models.

Cronbach’s alpha was estimated as a measure of internal consistency of the 12 survey items that specifically captured the extent of influence of contextual factors on implementing CVD interventions in LMIC (39, 40). Cronbach’s alpha ranged from 0 to 1 and a high Cronbach’s alpha value indicates a high level of internal consistency for the tool.

The EFA was conducted to determine how survey items, based on responses, were associated with the underlying construct of contextual influence on feasibility (40, 41). With the EFA, (a) clusters of survey items that were highly correlated with each other and identified with multiple dimensions of the survey were determined; (b) underlying constructs (i.e., the factors) identified were described; and (c) survey items that performed well in the clusters were identified as items to advance into the feasibility instrument. We applied orthogonal and oblique (promax) factor rotations to the EFA to test if factors were uncorrelated (orthogonal) or correlated, even if conceptually independent (oblique) (40, 41). Eigenvalues of factors were estimated to determine the factors that were responsible for the higher variance observed in item responses (42). Factors with greater eigenvalues are responsible for higher percentages of variance in item responses. For this study, we identified factors with an eigenvalue of 1 or higher (40, 42). The proposed instrument also had a good usability; given it was 12 items, which is within the quality range of 10 to 50 items (9, 18).

To determine the relative weights of these contextual factors on the feasibility of CVD-related EBI in LMIC, we estimated the correlation between respondents’ ratings of the quality or quantity of some inner and outer setting constructs and respondents’ average ranking of the construct’s influence on feasibility. A chi-square test was used to estimate statistically significant associations between the items connected to the quality and extent of influence of contextual factors.

Large language models—ChatGPT and Grammarly were used as supplementary tools, to refine grammar, confirm the interpretation of some of the written content, and reduce word count—at least a year post-study completion and authorship of the original manuscript versions.

## Results

### Item generation

#### Summary of systematic review data

The results from the qualitative content analysis showed a preponderance of data characterizing two inner-setting constructs and one outer-setting construct.

(Supplementary material 2). The CFIR inner setting constructs most described were (a) Readiness for Implementation, specifically the three subconstructs of “leadership engagement,” “available resources,” and “access to information and knowledge”; and (b) Implementation Climate, specifically the three subconstructs of “compatibility” (tangible fit), “relative priority,” and “goals and feedback.” The CFIR outer setting construct most represented was Patient Needs and Resources. The least documented CFIR construct was the outer setting construct of Peer Pressure. A detailed breakdown of reported characterizations of contextual facilitators and barriers to implementation feasibility of CVD interventions in LMIC and the associated CFIR contextual constructs are shown in Supplementary material 2.

#### Summary of key informant interview data

The three themes that emerged from the interviews were: (a) Invested leadership/stakeholder engagement process; (b) Grounded knowledge of individual and systems-level barriers and facilitators of optimal participation; and (c) Empowering models of engagement and training (see Supplementary material 3).

With the growing sense of ownership of these interventions, LMIC-based stakeholders (implementers and recipients alike) were more confident in the implementation process and benefits of the interventions and reported positive experiences about their involvement. Commentaries from key informants also provided depth to the range of contextual barriers and facilitators they encountered in their experiences implementing CVD interventions in LMIC (see Supplementary materials 4a,b).

**Figure 1:**
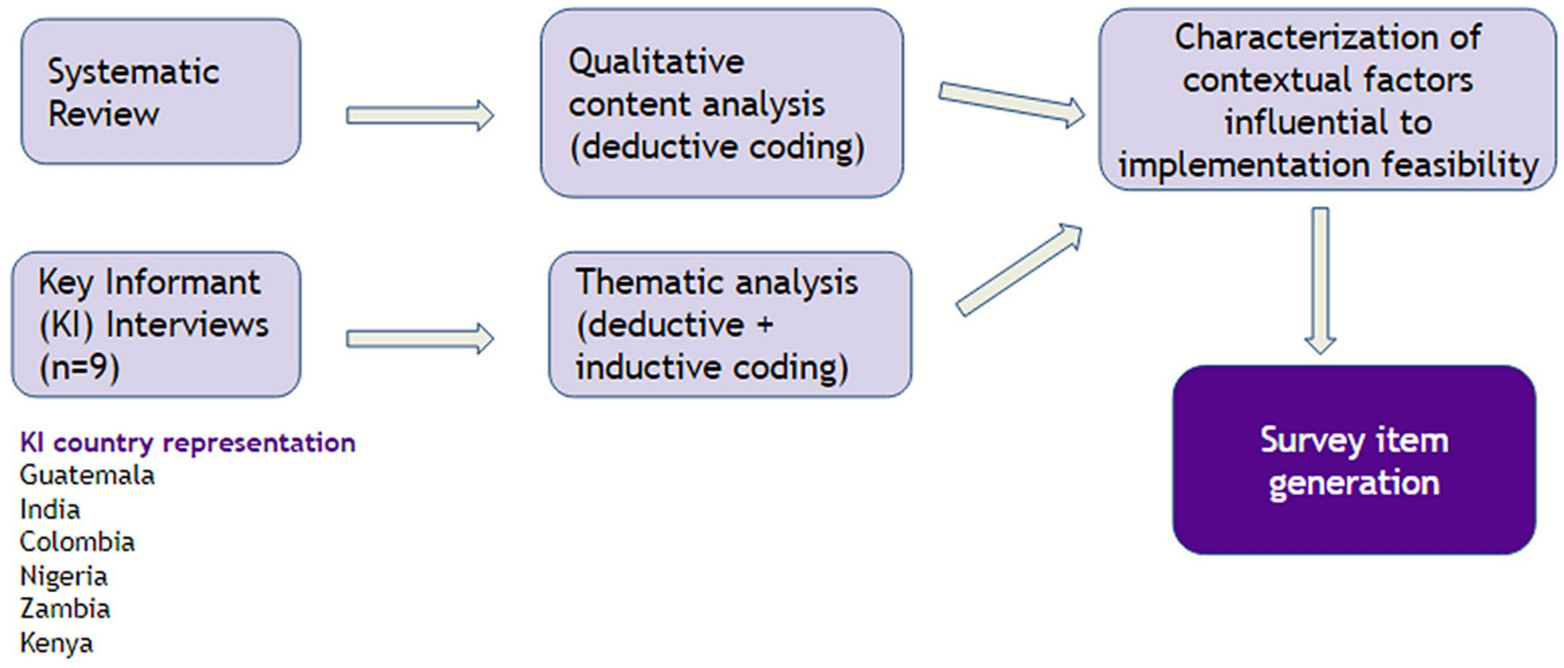
Analytical map for item generation using systematic review and key informant interview data.

Of the 66 survey items generated from the systematic review and key informant interviews, nine items came from two inductive codes identified from the key informant interviews, and 57 items were generated deductively from 16 CFIR inner setting and outer setting codes (see Supplementary material 5). The 66 items went through three rounds of iteration before advancing to item rating (see Figure 1).

### Item rating

Survey items were rated based on two qualities: relevance and importance. The first round of rating emerged with 42 items, where at least six of 13 raters had a positive agreement on both rating qualities. The second round of rating emerged with 17 items, where at least five raters had a positive agreement on both rating qualities. Two rounds of rating, using the Delphi method, produced a final list of 17 survey items (see Table 2).

**Table 2:** The final list of survey items (*n* = 17) from the Delphi-method item rating exercise, labeled with their corresponding CFIR or inductive codes.

Survey items	Response options
Conducting a needs assessment or contextual evaluation of our intervention setting before implementation (CID)	Not at all influential (1); A little influential (2); Moderately influential (3); Highly influential (4); Extremely influential (5)
Identifying implementation barriers before implementation (CID)	
Leveraging the existing workforce at intervention site(s) to deliver the intervention (IC^C^)	
The availability of physical infrastructure (SC)	
Presence of supportive community culture (C)	
Intervention’s implementation process fits into participants’ daily routine (IC^C^)	
Providing inclusive learning strategies (e.g., training, certification programs, workshops, consultations) to implementers (IC^LC^)	
Easy channels for providing feedback on the intervention (IC^GF^)	
Leaders’ and key stakeholders’ engagement and support for intervention (RI^LE^)	
Providing equipment for implementing the intervention (RI^AR^)	
Collaborating with community organizations for the intervention (Co)	
Providing training to implementers (RI^AR^)	
The interaction between the implementers and the beneficiaries of the intervention (NC)	Poor (1); Fair (2); Good (3); Very Good (4); Excellent (5)
The convenience of the intervention’s implementation process to implementers’ routine workflow (IC^C^)	
The implementers’ access to information and knowledge to optimally implement the intervention (RI^AKI^)	
In your opinion, how involved should leaders and key stakeholders be with implementing an intervention in a low- and middle-income country setting? (RI^LE^)	Not at all involved (1); A little involved (2); Moderately involved (3); Highly involved (4); Extremely involved (5)
In your opinion, how supportive are existing government policies towards implementing your intervention in this setting? (EPI)	Not at all supportive (1); A little supportive (2); Moderately supportive (3); Highly supportive (4); Extremely supportive (5)

CFIR and inductive codes.

Context for Intervention Design (CID); Implementation Climate (Compatibility) (IC^C^); Structural Characteristics (SC); Culture (C); Implementation Climate (Learning Climate) (IC^LC^); Implementation Climate (Goals and Feedback) (IC^GF^); Readiness for Implementation (Leadership Engagement) (RI^LE^); Readiness for Implementation (Available Resources) (RI^AR^); Cosmopolitanism (Co); Network and Communications (NC); Readiness for Implementation (Access to Knowledge and Information) (RI^AKI^); External Policies and Incentives (EPI).

### Survey building and pretesting

The final 28-item tool included the following structure: 15 CFIR-coded and two inductively coded survey items on contextual influence on the feasibility of CVD interventions, one survey item that measures agreement on the definition of feasibility as defined by Proctor and colleagues, one survey item that captures respondents’ self-reported perceived feasibility of their CVD interventions, and nine survey items that collected data on the nature of research/practice and intervention settings in which respondents were involved. These questions covered the number of years in research/implementation; respondents’ field of CVD research; country location; level of CVD prevention intervention administered (primary/secondary/tertiary); target population; duration of intervention in months; funding source; evidence source guiding interventions (WHO recommendations and literature evidence); and population setting (rural, peri-urban, urban).

Feedback from the retrospective cognitive tests covered issues around the framing of the survey questions, prompts, and the Qualtrics presentation style. This information was used to revise the survey. The survey was typically completed in 15 min.

#### Descriptive statistics of survey population

The survey population (*N* = 216) consisted mainly of CVD researchers (36.6%; 79/216), with 34 respondents who had multiple roles in CVD research and implementation. Approximately 45% (97/216) of respondents reported that literature evidence informed their intervention design and implementation, followed by 43% (92/216) of respondents who used WHO guidelines to inform their interventions. About 31% (69/216) of respondents reported using more than one source of evidence to inform their interventions. About 48% (103/216) of respondents implemented their interventions in rural settings, with 14.8% (32/216) of interventions occurring in more than one type of demographic setting. Most respondents (55.6%; 120/216) reported being involved in patient-level interventions, with 31.5% (68/216) of respondents reporting interventions that targeted more than one type of intervention recipient such as interventions targeting both patients and healthcare workers Most of the interventions (59.7%; 129/216) provided or supported the primary prevention of CVD, with 24.1% (52/216) of interventions addressing more than one dimension (primary, secondary, and tertiary) of CVD prevention. About 41% (90/216) of respondents received government funding for their work, and 16.2% (35/216) received funding from multiple sources besides the government. Respondents averaged 4.5 years (SD: 5.5 years) of CVD research or implementation experience, with their most recent intervention lasting an average of 16.3 months (SD: 17.8 months) (see Table 3).

**Table 3:** Descriptive statistics of survey population (*n* = 216).

Survey sample characteristics	*N* (%)
Role of respondent	
Researcher	79 (36.6)
Implementer	32 (14.8)
Health policymaker	20 (9.3)
Health administrator	18 (8.3)
Health practitioner	50 (23.1)
Other	57 (26.4)
At least 2 or more roles	34 (15.7)
Source of evidence informing intervention	
WHO guidelines	92 (42.6)
Literature evidence	97 (44.9)
Existing intervention	78 (36.1)
Other	15 (6.9)
2 or more sources	69 (30.6)
Intervention setting	
Rural	103 (47.7)
Peri-urban	48 (22.2)
Urban	78 (36.1)
Other	2 (0.9)
At least 2 types of setting	32 (14.8)
Intervention recipients	
Patients	120 (55.6)
Health workers	65 (30.1)
Health facilities	45 (20.8)
Population	57 (26.4)
Other	7 (3.2)
At least 2 types of recipients	68 (31.5)
Type of intervention	
Primary prevention	129 (59.7)
Secondary prevention	88 (40.7)
Tertiary prevention	36 (16.7)
Other	6 (2.8)
At least 2 types of intervention	52 (24.1)
Source of funding	
Government	90 (41.7)
Private for profit	19 (8.8)
Private nonprofit	69 (31.9)
Institutional	49 (22.7)
Other	11 (5.1)
At least 2 sources of funding	35 (16.2)
Average years of research or implementation experience, Mean (SD)	4.6 (5.5)
Average duration of intervention project in months, Mean (SD)	16.3 (17.8)

#### Country location data visualization

**Figure 2:**
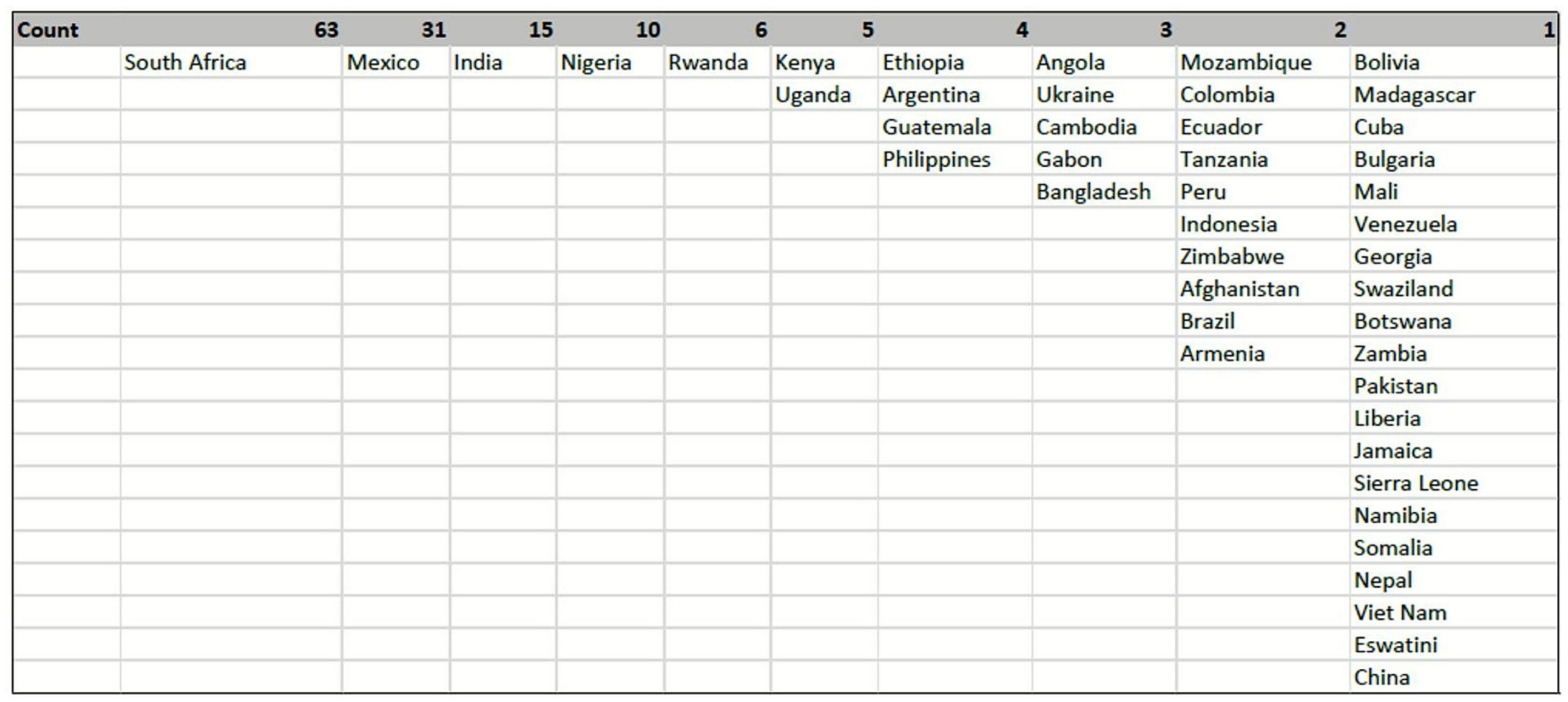
Geographical distribution of respondents CVD research and implementation experience in LMIC.

Respondents represented 46 unique LMIC, where the three most represented countries were South Africa (29.2%; 63/216), Mexico (14.5%; 31/216), and India (6.9%; 15/216). The sub-Saharan African region, with 20 countries represented, had the highest number of respondents (53.2%; 115/216) in the survey (see Figure 2).

#### Respondents’ agreement on the definition of feasibility and rating of intervention feasibility in LMIC

About 80% (172/216) of survey respondents agreed with Proctor and colleagues’ definition of feasibility as “the extent to which a new treatment or innovation can be successfully used or carried out within a given agency or setting (8).” Approximately 69% of the respondents (150/216) rated their most recent interventions as moderately to highly feasible.

#### Perceived extent of influence of contextual factors on implementation feasibility

Of the 17 CFIR-coded and inductively coded survey items, 12 items specifically captured the opinions of respondents on the extent of influence that contextual factors had on the feasibility of CVD interventions in LMIC. For all 12 items, 63.4% (137/216)—81.5% (176/216) of respondents, perceived that the contextual factor in each question had a high influence on the feasibility of implementing CVD interventions in LMIC (see Table 4).

**Table 4:** Response distribution on extent of contextual influence on feasibility of implementing CVD interventions in LMICs.

Survey item	Low to moderate contextual influence, *N* (%)	High contextual influence, *N* (%)
Conducting a needs assessment or contextual evaluation of our intervention setting prior to implementation	73 (33.8)	142 (65.7)
Identifying implementation barriers before implementation (pre-implementation)	58 (26.9)	158 (73.2)
Leveraging the existing workforce at intervention site(s) to deliver the intervention	73 (33.8)	143 (66.2)
The availability of physical infrastructure	77 (35.7)	139 (64.4)
Presence of supportive community culture	71 (32.9)	145 (67.1)
Intervention’s implementation process fits into participants’ daily routine	78 (36.1)	137 (63.4)
Providing inclusive learning strategies (e.g., training, certification programs, workshops, consultations) to implementers	62 (28.7)	154 (71.3)
Easy channels for providing feedback on the intervention	76 (35.2)	140 (64.8)
Leaders’ and key stakeholders’ engagement and support for intervention	59 (27.3)	157 (72.7)
Providing equipment for implementing the intervention	59 (27.3)	156 (72.2)
Collaborating with community organizations for the intervention	70 (32.4)	146 (67.6)
Providing training to implementers	40 (18.5)	176 (81.5)

The remaining five survey items asked respondents to rate the quality of:

- interaction between implementing stakeholders and those benefiting from the intervention
- the convenience of the implementation process to implementers’ routine workflow
- implementers’ access to information and knowledge to aid the implementation
- expected involvement of leaders and other key stakeholders
- support of existing policies for implementing an intervention in their settings

More respondents reported having a high quality of interaction between implementers and intervention beneficiaries (53.2%; 115/216); implementers’ access to useful information and knowledge to aid implementation (53.2%; 115/216); and an expectation of leaders and stakeholders to be highly involved in implementation efforts (78.7%; 170/216). On the other hand, more respondents reported having low to moderate quality (50.5%; 109/216) in the convenience of the implementation process to implementers’ routine workflow and low to moderate support (75.9%; 164/216) from existing government policies for their interventions in the LMIC settings (see Table 5).

**Table 5:** Distribution of responses to the quality of contextual factors deemed influential to the feasibility of implementing CVD interventions in LMICs.

Survey item	*N* (%)
The interaction between the implementers and the beneficiaries of the intervention	
Low to moderate quality	88 (40.7)
High quality	115 (53.2)
The convenience of the intervention’s implementation process to implementers’ routine workflow	
Low to moderate quality	109 (50.5)
High quality	94 (43.5)
The implementers’ access to information and knowledge to optimally implement the intervention	
Low to moderate quality	88 (40.7)
High quality	115 (53.2)
In your opinion, how involved should leaders and key stakeholders be with implementing an intervention in a low- and middle-income country setting?	
Low to moderate involvement	36 (16.7)
High involvement	170 (78.7)
In your opinion, how supportive are existing government policies towards implementing your intervention in this setting?	
Low to moderate support	164 (75.9)
High support	43 (19.9)

From the Delphi process, only 5 items emerged from the survey items that captured the extent of influence and respondents’ quality rating of a few CFIR-coded contextual factors. These items were leadership and stakeholder engagement (Readiness for Implementation); the convenience of the implementation process to implementer’s routine workflow (Implementation Climate); and access to inclusive learning programs to provide information and knowledge to support implementation (Implementation Climate, Readiness to Implementation). There was a significant association between the extent of influence the intervention’s implementation fit with participants’ daily routine and how convenient the implementation process was with the implementers’ routine workflow (*p* = 0.008); other associations between these 5 items were insignificant.

#### Assessing item reliability and factor identification

We assessed the reliability of survey items (*n* = 12) that captured the extent of influence contextual factors have on the feasibility of implementing CVD interventions in LMIC. Cronbach’s alpha for all 12 survey items was 0.88, and an average inter-item correlation coefficient of 0.39 out of 1, which falls within the recommended range of 0.15–0.50 (40) indicating that this group of 12 items were internally consistent.

Factor analysis and identification using EFA produced three factors, where the cutoff of Eigenvalue was set at 1 or greater. After the initial round of EFA, we used uniqueness (a measure of an item’s variance that is not shared with other items) to eliminate one survey item (‘influence of leadership and stakeholder engagement…’) as it had the highest uniqueness value of 0.57 out of 1 of all 12 items. The second round of EFA with 11 survey items produced three factors with Eigenvalues greater than 1 and collectively explaining 82.31% of the total variance observed in the analysis—a value improved upon after removing the item with the highest uniqueness (40). In the third round of EFA, where item uniqueness was employed as a criterion for elimination, there was no enhancement observed in the overall variance, as determined by the Eigenvalue cutoff set at 1.0. Consequently, we opted to adhere to the factors delineated in the preceding second round of EFA. We also set a cutoff of 0.30 or greater for the factor loading of an item, to qualify that item as belonging to a factor (see Tables 6, 7).

**Table 6:** Factor identification of items, assessing the extent of influence of contextual factors on implementation feasibility in LMICs.

Factor number	Eigenvalue	Number of items with factor loading greater than 0.3
Factor 1	3.07	11
Factor 2	1.25	3
Factor 3	1.54	8

**Table 7:** Clustering of survey items and corresponding CFIR or inductive codes by factors.

Factor/domain 1 [factor loading >0.3; *n* = 11] Factor name: global contextual influence on implementation feasibility	
Survey items	CFIR or inductive code
Conducting a needs assessment or contextual evaluation of our intervention setting prior to implementation	Context for intervention design
Identifying implementation barriers before implementation	Context for intervention design
Leveraging the existing workforce at intervention site(s) to deliver the intervention	Implementation climate (Compatibility)
The availability of physical infrastructure	Structural characteristics
Presence of supportive community culture	Culture
Intervention’s implementation process fits into participants’ daily routine	Implementation climate (compatibility)
Providing inclusive learning strategies (e.g., training, certification programs, workshops, consultations) to implementers	Implementation climate (learning climate)
Easy channels for providing feedback on the intervention	Implementation climate (goals and feedback)
Providing equipment for implementing the intervention	Readiness for implementation (available resources)
Collaborating with community organizations for the intervention	Cosmopolitanism
Providing training to implementers	Readiness for implementation (available resources)

Factor/domain 2 [factor loading >0.3; *n* = 3] Factor name: positive internal and external community involvement with personnel capacity	
Survey items	CFIR or inductive code
Presence of supportive community culture	Culture
Collaborating with community organizations for the intervention	Cosmopolitanism
Providing training to implementers	Readiness for implementation (available resources)

Factor/domain 3 [factor loading >0.3; *n* = 8] Factor name: implementation capacity building and fit, informed by existing need and barriers	
Survey items	CFIR or inductive code
Conducting a needs assessment or contextual evaluation of our intervention setting prior to implementation	Context for intervention design
Identifying implementation barriers before implementation	Context for intervention design
The availability of physical infrastructure	Structural characteristics
Intervention’s implementation process fits into participants’ daily routine	Implementation climate (compatibility)
Providing inclusive learning strategies (e.g., training, certification programs, workshops, consultations) to implementers	Implementation climate (learning climate)
Easy channels for providing feedback on the intervention	Implementation climate (goals and feedback)
Providing equipment for implementing the intervention	Readiness for implementation (available resources)
Providing training to implementers	Readiness for implementation (available resources)

We further performed an oblique rotation (promax) on the second round of EFA to see if the factors identified were correlated with each other (see Supplementary material 6). All three factors had strong correlation coefficients with each other; on a scale of 0 to 1, the correlation between (i) Factors 1 and 2 was 0.72, (ii) Factors 1 and 3 was 0.69, and (iii) Factors 2 and 3 was 0.73.

We also measured the reliability of each factor using Cronbach’s alpha. All three factors exhibited strong reliability scores ranging from 0.72–0.87 for unrotated factors and 0.68–0.74 for rotated factors and strong average inter-item correlation coefficients ranging from 0.39–0.46 (unrotated) and 0.49–0.59 (rotated) (see Table 8).

**Table 8:** Unrotated and rotated factor reliability estimates and corresponding average inter-item correlation.

Factor number	Cronbach’s alpha	Average inter-item correlation
Unrotated factor estimates		
Factor 1	0.87	0.39
Factor 2	0.72	0.46
Factor 3	0.84	0.40
Rotated factor estimates		
Factor 1	0.74	0.49
Factor 2	0.68	0.51
Factor 3	0.74	0.59

#### Bivariate analyses exploring associations between extent of contextual influence on feasibility and respondents’ rating of the feasibility of interventions

Independent variables consisted of four composite (continuous) variables described below:

- Composite 1 = Sum of 5-point Likert response scores of all 12 items capturing the extent of influence contextual factors have on feasibility. The scores ranged from 12 to 60 points.
- Composite 2 = Sum of scores of all 11 items under Factor 1, with scores ranging from 11 to 55 points.
- Composite 3 = Sum of scores of the 3 items under Factor 2, with scores ranging from 3 to 15 points.
- Composite 4 = Sum of scores of the 8 items under Factor 3, with scores ranging from 8 to 40 points.

The dependent (binary) variable was respondents’ rating of their interventions’ feasibility as Low feasibility-0 vs. Moderate to High feasibility-1. Four logistic regression models were conducted, and the results showed that with every point increase in Composite 1, there was a 6% increased odds of rating an intervention as moderately to highly feasible [OR: 1.06; *p* = 0.02; 95%CI = 1.01–1.11]. This result supported our hypothesis that contextual factors directly influence how researchers or implementers rate the feasibility of CVD interventions in LMIC (see Table 9). Composite scores from Factors 1 and 3 also showed statistically significant and positive association between the influence of contextual factors and feasibility of interventions.

**Table 9:** Bivariate logistic regression models for contextual influence and self-reported intervention feasibility rating (*n* = 173).

Regression model	Crude OR	*P*-value (95% CI)
Composite 1 (12-items)	1.06	0.02 (1.01–1.11)
Composite 2 (Factor 1)	1.06	0.02 (1.01–1.12)
Composite 3 (Factor 2)	1.17	0.06 (0.99–1.38)
Composite 4 (Factor 3)	1.09	0.02 (1.01–1.16)

## Discussion

We devised the CATALYTIC tool using the recommended methodologies outlined by experts for instrument design (28, 29, 43, 44). Comprised of 12 survey items, this tool adeptly encapsulates respondents’ perceptions regarding the contextual impact on the implementation feasibility of cardiovascular disease-related evidence-based interventions in LMIC (see Table 10). The survey data clearly demonstrates that contextual factors wield a significant influence on the feasibility of cardiovascular disease interventions in these regions, affirming the primary hypothesis of our study.

**Table 10:** The **C**ontextual Index of Fe**A**sibili**T**y on E**A**r**LY**-S**T**age **I**mplementation in LMI**C**s-CATALYTIC tool responses ranged from “Not at all influential (1)”, “A little influential (2)”, “Moderately influential (3)”, “Highly influential (4)”, and “Extremely influential (5)”.

Survey items
Conducting a needs assessment or contextual evaluation of our intervention setting prior to implementation (CID)
Identifying implementation barriers before implementation (CID)
Leveraging the existing workforce at intervention site(s) to deliver the intervention (IC^C^);
The availability of physical infrastructure (SC)
Presence of supportive community culture (C)
Intervention’s implementation process fits into participants’ daily routine (IC^C^)
Providing inclusive learning strategies (e.g., training, certification programs, workshops, consultations) to implementers (IC^LC^)
Easy channels for providing feedback on the intervention (IC^GF^)
Leaders’ and key stakeholders’ engagement and support for intervention (RI^LE^)
Providing equipment for implementing the intervention (RI^AR^)
Collaborating with community organizations for the intervention (Co)
Providing training to implementers (RI^AR^)

CFIR and inductive codes.

Context for Intervention Design (CID); Implementation Climate (Compatibility) (IC^C^); Structural Characteristics (SC); Culture (C); Implementation Climate (Learning Climate) (IC^LC^); Implementation Climate (Goals and Feedback) (IC^GF^); Readiness for Implementation (Leadership Engagement) (RI^LE^); Readiness for Implementation (Available Resources) (RI^AR^); Cosmopolitanism (Co).

What sets our new tool apart is its unique capability to furnish a composite score derived from high-priority contextual factors, thereby enabling a comprehensive assessment of intervention feasibility in diverse LMIC. Unlike existing feasibility tools like the FIM, our tool offers heightened visibility into the intricate real-world factors that underpin both feasible and successful implementation. It excels in capturing nuanced contexts within its measure, a facet where other tools like the FIM may fall short (45).

A strength of our methodology lies in the diversity encapsulated within our survey respondents, comprising a wide-ranging cohort of cardiovascular disease researchers and implementers hailing from 46 countries within LMIC. Their collective experiences and perspectives serve as invaluable sources of insight, offering pertinent and multifaceted understandings of how contextual factors intricately shape the landscape of implementation feasibility in these countries. Using this approach, we were able to characterize Implementation Climate and Readiness for Implementation and their subconstructs, which were predominant during the exploratory phase of this study and in the survey data, indicating their high relevance to researchers and implementers, among inner setting and outer setting constructs of the CFIR. This highlights respondents prioritizing a selection of contextual factors that seems more tangible and functional to their real-world experience of implementing CVD interventions, against a backdrop of limited resources in LMIC (4).

The development of the CATALYTIC tool is significant as its implementation promises to pave the way for a notably enhanced and precise evaluation of feasibility, particularly within settings where the interplay of environmental and organizational factors assumes a pivotal role in determining the viability of implementing evidence-based interventions in LMIC (2–4). The CATALYTIC tool stands out in its capacity to bridge a critical gap by actively addressing the pressing necessity for extensive cross-cultural validation of instruments. This urgency stems from the evident scarcity of implementation outcome instruments specifically tailored to the unique contexts of LMIC, a gap starkly highlighted in previous reviews (9, 46). It is worth highlighting that a 2020 review examining implementation outcome instruments within physical healthcare settings specifically identified a mere three instruments designed for LMIC. Remarkably, none of these instruments were focused on measuring implementation feasibility, signaling a substantial gap in addressing this crucial aspect within such contexts (46).

The psychometric evaluation of the CATALYTIC tool has yielded notably promising results, showcasing its robustness and reliability. Evidencing its strength, the tool boasts an impressive reliability score, measured by Cronbach’s alpha, standing at a commendable 0.88 out of 1.00 (40). Furthermore, a meticulous three-factor exploratory factor analysis (EFA) has underscored its reliability by distinctly delineating three factors: the overarching influence of contextual elements on implementation, the fostering of positive community engagement intertwined with personnel capacity, and the nuanced consideration of implementation capacity building and alignment with existing needs and barriers. The use of uniqueness value during the EFA to eliminate specific items that lowered the relevance of other items, speaks to the rigorous psychometric evaluation of the tool (47).

Moreover, the tool’s credentials are bolstered by its strong content validity, stemming from the Delphi method employed for item prioritization and selection. This method not only shaped the tool’s construction but concurrently validated the content of its survey items. This dual role underscores its efficacy not just as a measurement instrument but also as a tool integral to the strategic design of psychometrically sound assessments (30, 48). This confluence of reliability and content validity positions the CATALYTIC tool as a robust and comprehensive psychometric instrument tailored for intricate assessments within implementation science.

In healthcare settings, the Feasibility of Intervention Measure (FIM) is currently used to assess feasibility. However, the FIM was not designed to address any specific purpose, persons, or situation (45). Moreover, there is no evidence that it is applicable for implementation in LMIC, given that the FIM has not been widely validated across different LMIC settings. While LMIC-based researchers persist in their efforts to adapt the FIM for assessing intervention feasibility, Dorsey et al. (49), Salisbury et al. (50), and Wright et al. (51) the advent of the CATALYTIC tool marks a significant stride within the field of implementation science. This tool offers a tailored solution that more accurately encapsulates the intricacies of implementation feasibility within the dynamic landscape of LMIC. Its application holds the potential to guide endeavors aimed at addressing the limited presence or absence of contextual factors during implementation, propelling beneficial health interventions beyond mere pilot phases (27). This index tool is also the first of its kind to capture the influence of select contextual factors on the feasible implementation of cardiovascular health initiatives and can be adapted for programs addressing a range of disease types/health conditions in a resource-challenged context.

Future research will involve validating the tool’s ability to capture the concept of contextual influence on implementation feasibility (52, 53). This validation process will encompass several facets, including construct validity assessments aimed at gauging its correlation with other variables known to influence implementation feasibility. For instance, examining its relationship with the CFIR Intervention Characteristics subconstruct ‘complexity—perceived implementation difficulty (4, 52, 53) will form a crucial part of this validation process. Additionally, the tool’s validation will entail evaluating its performance using data from feasibility studies that have measured recommended metrics for assessing feasibility. Parameters such as the level of intervention demand, acceptability, implementer engagement, and resource availability will be considered (26, 54). Another avenue for construct validity assessment involves scrutinizing the tool’s association with indicators of early-stage implementation outcomes, such as appropriateness and acceptability, which are empirically interconnected with feasibility (8, 45). This multifaceted validation approach aims to comprehensively affirm the tool’s robustness in effectively capturing and correlating with various facets of implementation feasibility.

### Strengths and limitations

Our survey development process was meticulously executed, focusing on fortifying the psychometric robustness of the survey items to effectively evaluate the CFIR-derived contextual impact on the feasibility of CVD interventions in LMIC. This involved a methodical approach, utilizing CFIR contextual domains to steer our literature search and analyze key informant interviews. By anchoring all surveyed items to available evidence, we ensured their alignment with established knowledge.

The inception of the CATALYTIC tool was the result of active engagement with a diverse spectrum of experts in CVD research and implementation within LMIC settings. Their collective expertise was instrumental in crafting a tool that authentically mirrors their perspectives on how contextual elements significantly shape the practical execution of feasible CVD interventions within LMIC.

Throughout the Delphi method’s two rounds, participants highlighted the time-intensive nature of the process, which inevitably affected participant retention, leading to dropouts or loss to follow-up—a characteristic not uncommon in Delphi methodologies (29). Despite employing a census approach, practical limitations, such as online administration constraints and limited outreach, posed challenges in engaging all eligible respondents. These limitations potentially resulted in gaps regarding country representations, language barriers, and technological constraints, impacting the inclusivity of our participant pool.

## Conclusion

The CATALYTIC tool represents an important step towards evaluating the implementation feasibility of CVD interventions within LMIC. Specifically designed to capture the intricate web of contextual factors impacting feasibility, this tool establishes a standardized framework for consistently measuring and monitoring feasibility metrics. Through comprehensive survey analysis, it has pinpointed high-priority contextual factors that significantly shape how researchers and implementers perceive the feasibility of implementing CVD interventions in LMIC.

Beyond its immediate application in CVD interventions, the adaptability of this tool to diverse health conditions within resource-constrained settings stands as a testament to its versatility. Its potential extends far beyond mere assessment; the tool holds the capacity to inform priority setting, guide decision-making processes, and streamline resource allocation efforts across various health domains. By facilitating a more nuanced understanding of contextual influences, its widespread application could catalyze the advancement of CVD interventions and ultimately foster improved health outcomes within these resource-challenged settings.

## Data Availability

The original contributions presented in the study are included in the article/Supplementary material, further inquiries can be directed to the corresponding author.
