# Supplementary material for: The CATALYTIC tool to assess feasibility of implementing evidence-based interventions for cardiovascular diseases in 46 low- and middle-income countries: survey outcomes and tool reliability testing
Source: Front Public Health. 2025 Dec 10;13:1597996. doi: 10.3389/fpubh.2025.1597996 (PMC12727921; doi:10.3389/fpubh.2025.1597996)
Supplement: Supplementary file 1 [file Table_1.docx]

**Supplement 1**

Below is a list of member countries on NIH-Fogarty, GRIT, GACD, and PASCAR.

- NIH-Fogarty International Center (28 countries): Argentina, Azerbaijan, Bangladesh, Bolivia, Botswana, Costa Rica, Ethiopia, Georgia, Ghana, India, Indonesia, Jamaica, Kenya, Kosovo, Lebanon, Malawi, Mongolia, Nepal, Nigeria, Pakistan, Peru, South Africa, Tajikistan, Tanzania, Thailand, Turkey, Uganda, and Vietnam.
- GRIT (8 LMIC members): Ghana, Guatemala, India, Kenya, Malawi, Nepal, Rwanda, and Vietnam.
- GACD (31 LMICs with CVD-related interventions): Argentina, Bangladesh, Brazil, Bulgaria, Cambodia, Chile, China, Colombia, Ecuador, Eswatini, Fiji, Ghana, India, Indonesia, Kenya, Malawi, Malaysia, Mexico, Myanmar, Nepal, Nigeria, Pakistan, Peru, Philippines, Rwanda, Samoa, South Africa, Sri Lanka, Tanzania, Uganda, and Vietnam.
- PASCAR (25 member countries, all situated in Africa): Algeria, Angola, Benin, Burundi, Cameroon, Cote d’Ivoire, Democratic Republic of Congo, Egypt, Ethiopia, Gabon, Ghana, Kenya, Mauritania, Mauritius, Morocco, Mozambique, Niger, Nigeria, Senegal, South Africa, Rwanda, Sudan, Tanzania, Tunisia, and Uganda.
