# Supplementary material for: The CATALYTIC tool to assess feasibility of implementing evidence-based interventions for cardiovascular diseases in 46 low- and middle-income countries: survey outcomes and tool reliability testing
Source: Front Public Health. 2025 Dec 10;13:1597996. doi: 10.3389/fpubh.2025.1597996 (PMC12727921; doi:10.3389/fpubh.2025.1597996)
Supplement: Supplementary file 2 [file Table_2.docx]

**Supplement 2.** Characterization of contextual factors affecting the feasibility of CVD interventions in LMICs mapped to CFIR’s 16 contextual constructs and sub-constructs

| **Construct or sub-construct Name** | **Facilitators** | **Barriers** |
| --- | --- | --- |
| **Inner Settings** | | |
| Structural Characteristics | Supportive management structure of existing intervention location for interventions; familiar hospital setting enhanced implementers’ access to recipients’ information and logistical ease; improved healthcare infrastructure; organizational upgrade to accommodate intervention; improved environmental setting; administrative accommodation for implementers; existing health centers that are accessible, staffed and equipped**;** equality in living standards | Poor physical environment**;** limited infrastructural capacity; wide geographic dispersion of intervention sites; suboptimal staffing; lack of/limited technical expertise and support for information technology needs; drug distribution challenges telecommunication challenges (low computerization); uncertain knowledge of health system; remote locations**;** potential misuse of incentive-based programs in resource constraint settings |
| Network and Communications | Trust-building through informal discussions, social gatherings, and dedicated time commitment with CHWs; building familiarity with recipients before intervention start; existing social activities, support from community leaders, and internal site team activity; repeated consultation workshops; respectful interactions between implementers and recipients; close coordination between implementers and patients’ physicians; existing social activities; holding coordination meetings with stakeholders; patient-empowered and respectful communication with nurses/health providers; communications using local dialects; effective communication strategy and plan | Lack of communication from supporting facilities; language barriers; lapse in communication between providers and patients; lack of existing internal site team activity; suboptimal patient-provider communication/unreliable means of communication with participants; lack of communication within different cadres of health providers and implementers |
| Culture | Use of community empowerment approach; culturally appropriate/compatible implementation; shared culture between researchers and implementers; positive attitudes of implementers; operating in teams; implementers’ confidence in employing implementation tools; prior experience with implementation tools; operating in teams | Lack of community support; lack of parental involvement; prevailing poor social norms regarding risk factors for NCDs; spirituality/faith-based avoidance of intervention; stigma of disability; cultural and language barriers; cultural belief in alternative interventions; unprofessionalism; unhealthy food culture and preferences; patients’ preference for traditional medicine over western medicine; culturally rooted gender differences |
| **Implementation Climate sub-constructs** |  | |
| Tension for Change: | General interest of individuals in learning more about CVD condition; high disease burden and prevalence; high demand for intervention; recommendation for regular follow-ups; enhancement in quality and relevance of skills performed by health providers; providers’ confidence in prescribing the intervention; recommendations of intervention components by implementers; high implementers’ acceptability/support for intervention; high uptake by implementers; higher uptake among implementers with prior experience with an older version of intervention guidelines; preexisting accessibility to services offered by intervention; greater social unacceptability for negative health behavior (smoking); high prioritization for tackling CVD and other NCDs by stakeholders; high prioritization for tackling CVD and other NCDs by stakeholders | Providers hesitant in encouraging recipients to take intervention; more focus on communicable/maternal health diseases; disease not perceived as life-threatening; lack of awareness for need/benefit of intervention; continued high prevalence of harmful health behavior; implementers’ objection to new intervention component (incentive-based vs. fixed remuneration); lack of awareness for intervention need; lack of knowledge about the disease and its therapy |
| Compatibility | Leveraging existing network of personnel and gatekeepers to implement and monitor interventions; relevance of topics suited to implementers’ roles; implementers’ ease of access to patients’ records and logistic convenience; intervention implemented as close to real-world conditions (e.g., not receiving certain intervention components for free); consideration for implementers’ existing work hours and workload; convenient meeting locations; adequate staffing; integration of intervention implementation into existing healthcare routine and workflow; choice in selecting service priorities to implement; easy and integrative intervention components for implementers’ workflow/recipients’ routine living; convenience accessing location and timing of intervention by participants; affordable intervention; mobile technology support for CVD management services, monitoring, and patient reminders; pragmatic intervention design; adaptation (language) of intervention; provider hands-on involvement in intervention design and planning; customized information delivery; adjusting education strategies, topics, and printed materials to community/national needs, preferences, and guidelines; easy and integrative intervention components in recipients’ routine primary care practice; intensification of skills for existing health providers to align with intervention goals | Lack/inconvenient/lengthy timing; too many implementation sessions; overburden in learning and execution of intervention on existing workforce; intervention not factored into the workflow; high staff turnover; inconvenient/not dedicating sufficient time to training; limited organizational capability; existing implementers' workload; the overwhelming amount of effort and time needed for patient education; time constraints for patient consultations; resistance to implementing intervention workflow; high staff turnover; invalidating community health workers referrals |
| Relative Priority | Positive rating of educational content for patients’ training; formative research to establish target population’s priority; positive perception of intervention value among patients and providers; participants’ willingness to recommend intervention; positive support/enthusiasm/value added of intervention from implementers; perceived usefulness of training content; adoption of intervention by healthcare providers/facilities; confidence of implementers and leaders in intervention; enthusiasm for training opportunities; implementers’ engagement in iterative tool/intervention development; willingness to participate/compliance; public support for intervention; implementers/expert stakeholders saw the potential value of the intervention; high acceptability of intervention; leadership satisfaction with intervention; participant willingness to engage in intervention; providers’ confidence in intervention; uptake of the intervention | Competing priorities with gainful employment; competing basic priorities of recipients; participants’ lack of trust in intervention; participants’ reluctance to engage in intervention/low uptake of intervention components (do not see the need for); participants’ preference for curative methods over management of the disease; preferential belief in traditional health practices over orthodox western health interventions; implementers’ reluctance to engage in/promote intervention; implementers’ overemphasis on curative over preventive measures; implementers' lack of satisfaction/support with intervention tool (tech); more focus on treatment over preventive health practice; providers’ concern on intervention side effects; more emphasis on communicable diseases; low uptake for intervention because of perceived lack of need; delayed adoption of interventions; competing priorities and resources dedicated to them |
| Organizational Incentives and Rewards | Certification of implementers; provision of incentives to implementers; incentives for implementers; providing certifications for implementers; perceived increase in respect for nonclinical health workers due to training; consistent institutional and financial support; compensation for additional services | Insufficient and delayed remuneration for implementers; discouraging/lack of (financial) incentives for implementers |
| Goals and Feedback | Collaborators’ and researchers’ openness to learn during the implementation process and flexibility to change based on new knowledge; organic feedback from trainees/implementers on target recipients, delivery and intensity of intervention; intervention components rated as positive and helpful; broad satisfaction with intervention frequency and length; good acceptability of intervention; provider engagement with feedback channels; sufficient feedback activities; implementers’ feedback for refresher training courses; implementers’ high positive rating on usefulness of training and education; agile design with early end-user feedback; intervention delivery inspired recipients’ control of their health; intervention delivery was inclusive of close relations to recipients; regular research visits and interviews with key personnel for feedback on needs and willingness to participate in study; continued engagement of stakeholder to further adapt intervention; implementers provided feedback on intervention’s good usability and usefulness; researchers acted on implementers’ and participants’ feedback; good communication and support for communicative medium (SMS) between participants and implementers (incorporated family in intervention); used feedback from stakeholders/participants and evaluation to revise and target intervention; positive feedback from participants; routine communication between implementer and participant; patient satisfaction | Lack of reminders; lack of feedback from participating facilities; feedback on the burden of caregiver and care time and effort involved; patients’ feedback on misperceptions as being noncompliant; patient feedback on poor treatment from health providers; low level of satisfaction with intervention target; feedback on the intervention being difficult to adhere; lack of feedback from supporting facilities; no formal monitoring and evaluation component s of intervention |
| Learning Climate | Collaborators’ and researchers’ openness to learning during the implementation process and flexibility to change based on new knowledge; participative model of training the g; the value of patient rating of providers; joint implementer-recipient intervention building; use of implementers with prior qualification, training, and experience; bidirectional learning, dialog, and ongoing reflection related to project implementation; support from trainers and research team; build local and diverse research capacity; experts collaborated with participants; quality assessment of implementation fidelity; supervision of implementation; process evaluation/tracking |  |
| **Readiness for Implementation sub-constructs** |  | |
| Leadership Engagement | Interest, buy-in, support, partnerships, and ownership from leadership at multiple levels of government and stakeholders; commitment from political leaders, and strong leadership and governance; engagement through community advisory boards; good community engagement and trust; decision makers’ support/commitment for program implementation; decision makers’ knowledge of intervention; defining clear roles for leadership and advisory boards; consistent institutional support; political readiness; effective management | Lack of leadership cooperation; lack of leadership support; lack of support from designated supervisors within the existing health system; unavailability of government stakeholders in intervention design; challenge identifying suitable government stakeholders to engage; undefined roles for local leadership; limited/lack of leadership engagement/familiarity with intervention; leadership/management turnover; absence of key stakeholders from meetings; different stakeholder expectation; low level of political will |
| Available Resources | Provision of free medications and services as part of the intervention; training of implementers; increase in available resources, and equipment; patient education materials; affordable services; proposed adequate equipment and staffing; available space and time; availability of local resource personnel for added support; continued training, communication, and programmatic support; consistent institutional and financial support; access to data; data assessment capacity; training aids and manuals; tech resources to support intervention; access to healthy food | Lack of public space to host intervention; inconsistent/lack of supply of medication; unavailable/inadequate resources; lack of/limited financial resources; equipment breakdown/no equipment; insufficient hospital beds; insufficient/inconvenient timing for recipients/implementers; unavailability of medications; limited resources (space, electricity, lab); limited transportation for implementers; insufficient/limited training (quantity and time); retrenchment of implementers due to funding cut; competing scarce resources; lengthy training time; inadequate funding and donor overdependence; broken/lost and other operational challenges of intervention tools |
| Access to Information and Knowledge | Adequate training provided to implementers, with an empowering approach applied in certain scenarios; delivery of intervention information using computerized systems; electronic and easy use of SMS for scheduling and tailoring education/intervention; increased patient education and awareness of CVD conditions and intervention; standardized and detailed instructions for patients; high awareness of intervention among health providers; telemedicine; easy-to-use and understand educational/documentation materials; supervision/oversight/support; implementers’ retention of knowledge and skills; positive rating of training and education; implementers’ access to expert consultation and troubleshooting; perceived recipients’ motivation to learn and incorporate intervention; relevant disease content covered in training; implementers’ ease of access to patient’s records and logistic convenience; use of software and electronic record; access to experts for knowledge and consultation; providing recipients needed orientation for intervention activities; integrated use of instruction, modeling, and practice to help participants achieve intervention goals; use of technical expertise | (Limited) existing noncommunicable disease training curriculum in medical institutions; low health education for patients; lack of awareness/incomplete knowledge of intervention; insufficient time to cover curriculum; omission in assessment for adequate training and knowledge uptake; lack of supervision; training gaps; difficulty in understanding intervention components; complexity in training implementers on multiple contents; reliance on suboptimal reporting systems/limited documentation and reporting tools; recipients’ self-reporting and inconsistencies; limited numbers of disease experts/specialists; lack of/limited technical expertise and support for IT needs; challenges in delivery optimal training with hands-on practice |
| **Outer Settings** | | |
| Patient Needs and Resources | Intervention component designed to integrate with preferred lifestyle; community/familial support; mobile app integrated into an intervention to eliminate barriers; provider routine engagement with patients to maintain retention; negotiable terms of intervention; easy-to-access intervention and services; matching interventions to residents’ health priorities; high education level; highly personalized intervention and implementation strategy; access to technology/internet; peer mentoring; affordable and accessible medications and services; ease of transportation; easy-to-use intervention tools | Patient-level challenges of transportation to clinics and access to medications; low participant turnout, inconvenient timing, and loss of income due to intervention activities. financial and emotional pressure; costly/unaffordable services and change in lifestyle; lack of medications; caregiver burden; patients have unreliable communication outlets; lack of insurance coverage; remoteness of locations; competing for basic priorities; financial constraints |
| Cosmopolitanism | Partnerships with local government bodies, research institutions, and community organizations; community engagement; working with trustworthy community organizations; mechanisms and collaboration in bringing scientific evidence to policymakers; public‒private/multilateral partnerships; new program promotion in partner companies; implementation partnerships; bilateral country collaboration | Limited community collaborations with need expertise and capacity; alternative private care providers |
| Peer Pressure | Not identified | Concurrent similar intervention campaigns skewing expected numbers of outcomes; lack of participation of women groups; lack of active NCD groups in setting |
| External Policies and Incentives | Aligned intervention to established guidelines; support from political leadership; supportive government policies; support from policymakers; provision of incentives for implementers; acquire proper documentation from a government agency; visible and national program dissemination; bilateral country collaborations; policy endorsement; health-progressive policy, ratification; donor funding; public‒private partnership; vertical or horizontal integrative style of leadership | Lack of compensation for additional workload; poor socioeconomic conditions; nonexistent government health promotion programs; nonexistent government training on disease management; technological challenges: countries’ low bandwidth, slow connections, and high service charges; complex interaction of psychosocial stressors and health service problems; insufficient and delayed remuneration for implementers; disruptive government policies, community or family influences; labor strikes; global sporting events; misaligned national policies; politics; natural disasters; smoking policy-to-impact misfit with no positive impact; limited insurance coverage; delay in policy and environmental intervention; no reason for policy implementation; lack of political will; absence of a national stroke clinical guideline, a national framework for quality improvement interventions for stroke |
