# Supplementary material for: The CATALYTIC tool to assess feasibility of implementing evidence-based interventions for cardiovascular diseases in 46 low- and middle-income countries: survey outcomes and tool reliability testing
Source: Front Public Health. 2025 Dec 10;13:1597996. doi: 10.3389/fpubh.2025.1597996 (PMC12727921; doi:10.3389/fpubh.2025.1597996)
Supplement: Supplementary file 3 [file Table_3.docx]

**Supplement 3: Key informant interview themes and characterizing quotes**

| **Theme** | **Summary** | **Quote** |
| --- | --- | --- |
| **Invested Leadership/Stakeholder engagement Process** | According to key informants, early and intentional leadership/stakeholder engagement made the implementation process more feasible, with the added benefit of helping research teams build legitimacy and trust with the organizations, communities, and locally based stakeholders with whom and for whom they are implementing the interventions. | *“What I learned from these two projects is that the planning process is need[ed], [and] make sure that you put in your time..., when you plan a proposal, plan enough time to do this. In addition, it is quite important to this process because then at the same time, you are empowering all people who are going to help you to do the intervention, either with the Ministry of Education and schools or the Ministry of Health and the health posts. So you start, like, well, “Let’s do this together, let’s work together and make best intervention for what you’re doing.” ... [By doing this], we are doing a better job knowing the context and interacting with people in the context*.” (KI 8)  *“Therefore, we had to do a couple of workshops with [stakeholders] to come to a common understanding of how we are defining healthy food and how they are defining. Therefore, it took some time to come up [to] a common ground.... Therefore, we had to have conversation with each and every group; it was time-consuming, but just to keep them all in this on the same pace and not let their internal conflict come to front or like affect the intervention.” (KI 2)*  *“We essentially invited representatives from each of this [stakeholder groups], care providers, patients with hypertension, patients who serve as peers in other chronic diseases like HIV peers, ourselves as investigators and researchers, and clinicians as well in that role that we also played. In addition, we had a human-centered design process where we, we took what we had at baseline and sort of tried to enrich it to make sure that the intervention was as closely aligned to what the problem was as possible.... We started off with the head of the noncommunicable disease division at the Ministry of Health, introduced the study to him; he was one of the supporters [of] the grant submission. Therefore, with his support, we’re able to go to the county level and [tell them] what we were hoping to do. Once they gave us permission, we go down with that permission to the subcounties, all the way to the lowest level. In addition, yes, and the good thing with the community entry is, through that path, we start to identify key program leaders, key clinicians, and members, like health management teams, and key community voices who we will need to talk to when we’re starting to do a baseline contextual evaluation.” (KI 4)* |
| **Grounded knowledge of individual and Systems-level Barriers and Facilitators of Optimal Participation** | Key informants emphasized a formative learning process through which researchers became familiar with the environment and population for whom they were implementing CVD interventions. They also highlighted the value of being flexible with international design and implementation process as this allowed for the interventions to be more feasible in their LMIC settings. | *“We went into essentially a formative evaluation, where we talked to everyone again, trying to just determine from the perspective of the community, from the perspective of patients with hypertension, from the perspective of clinicians and program administrators what they think the gaps are, as far as referral care for hypertension is. Do they think an intervention that combined health information technology and if using peers would be appropriate to address those issues? And if so, in what way do they think those things would need to be aligned? Once [we] have all th [is information], then the next thing (because all this is toward preparation) getting specific voices from all these groups together to come up with a unified refined intervention. Then, after that was a pilot phase, then the evaluation of the pilot, then finally we start we implemented the study.” (KI 4)*  *“We were also pretty flexible in making changes during the intervention period as well. Therefore, I think that was also like we [didn’t insist on] 100% of our [intervention design]. Therefore, we were also like flexible in making changes. In addition, we had defined like a minimum requirement, like 50% of the food should be whole grains—things like that. However, we were also flexible: If people were truly demanding more white bread, we would keep it, but it won’t be on the counter or something, it would be like, completely hidden, [but they were given the option to choose the existing unhealthy option]. Therefore, I think the flexibility of the intervention helped.” (KI 2)* |
| **Empowering Models of Engagement and Training** | Key informants agreed that engaging locally based stakeholders and implementers, with the aim to improve local ownership of CVD intervention efforts, empowered all parties involved. This was reflected using empowering models such as ‘Human centered Design, which places targeted intervention recipients at the core of how the intervention is designed and implemented. Another approach to promote ownership was also being negotiable with the terms of intervention delivery and participation. This outcome of empowerment was particularly poignant when researchers engaged existing networks of health workers and peers in implementing interventions in their communities and organizations. Part of that empowering engagement was reflected in the ways implementers were trained to administer the interventions. | *“You have to put enough time to train the whole staff that is going to be part of this and in that training, it is important that they feel like part of the project and not just someone who is receiving orders on how to do them. In addition, it’s important that you give opportunity to them to listen to the experiences of others. Therefore, we have these community advisory boards throughout the study, and they meet every six months. However, the idea at the beginning was that they meet every six months, and they can hear experience of the other health districts, the other auxiliary nurses—what they’re doing, how they’re doing it to make sure that the medicines are delivered to a patient for example.” (KI 8)*  *“Another major factor was our research staff. They were on it! They were like—monitoring every week. We had a feedback session with the chefs and intervention group every month. Therefore, I think it was also continuously monitored. Therefore, I think that was also one of the major facilitators.” (KI 2)* |
