# Supplementary material for: The CATALYTIC tool to assess feasibility of implementing evidence-based interventions for cardiovascular diseases in 46 low- and middle-income countries: survey outcomes and tool reliability testing
Source: Front Public Health. 2025 Dec 10;13:1597996. doi: 10.3389/fpubh.2025.1597996 (PMC12727921; doi:10.3389/fpubh.2025.1597996)
Supplement: Supplementary file 4 [file Table_4.docx]

**Supplement 4a.** CFIR- and non-CFIR-mapped contextual barriers to implementation feasibility in LMICs

| **CFIR Inner and Outer Settings Constructs**  **Domain/Construct/Sub-construct** | **Contextual Barriers** | **Illuminating Statements from Key Informants** | **Solutions to Barriers** | **Illuminating Statements from Key Informants** |
| --- | --- | --- | --- | --- |
| Inner Setting/Structural Characteristics | Overburdened health systems and the double burden of disease | “The CVD rates are rising for sure, but we still have unresolved issues of maternal-child health, nutrition, communicable diseases, and many others, right? And the primary healthcare system must deal with all of them at one go.” (KI 1) | Simplifying intervention process | “Therefore, the more complicated we make any of our programs, the more things the chap has to remember and report or write down, the less likely that would be done.” (KI 1) |
| Outer Setting/External Policies and Incentives | Negotiating power for reducing med costs is held by private health insurance companies that are profit-driven | “It is the insurance companies that maintain most of the health facilities. Therefore, it is one of the problems because while we talk about primary prevention, they are not all that interested in helping public health. The Health Ministry is not the owner of the health facilities; the health facilities are from the insurance companies.” (KI 7) | --- |  |
| Inner Setting/Culture | Pushback from professional bodies on the focus and strategy of the intervention    Pushback and organizational internal politics | “I told you about this pushback from a specific group within the organization. Therefore, we were working with a particular group that is more influential and another minority group felt the need to sabotage and they kind of wanted the intervention to fail. Therefore, we saw this kind of politics in another worksite in the industrial setting, where they were real political groups, different union groups of employees.” (KI 2) | Conversations, negotiations, and going over to a higher authority    Staying flexible while maintaining a minimum standard for operating | “I think as a researcher, we also did a couple of compromises with the kind of changes that we wanted to do. We did a couple of modifications to the recipe rather than taking whole items out. For example, instant noodles are very, very popular in Nepal. In addition, it’s not healthy, it also has trans fats and trans-fat is still not banned by the government. Therefore, we came to a conclusion like, whenever the cafeteria serves instant noodles, it will not serve the full serving, but it will cut it to half and add lentils.” (KI 2) |
| Inner Setting/Structural Characteristics    Inner Setting/Implementation Climate/Compatibility    Outer Setting  /External Policies  and Incentives | Poorly positioned drug supply management systems; coupled with poor technical coordination    Misaligned essential list of medications from national experts and state government, causing procurement challenges | “Most of the primary health care system doesn’t have a laboratory network for it, for instance, right?... Having a standard program for diabetes is impossible without having a good basic quality lab test for glucose.” (KI 1)  “For example, drug supply management is the major issue…. The medications were not available, or it was insufficient or inadequate or ill distributed.” (KI 1)  “Like there are so many challenges concerning drugs, for example, one is supply chain management. The state government decided on certain drugs for their protocols, right… Those are the medications to be provided timely in the healthcare sector, but the essential list doesn’t include [them] and they do not bring them at all. --Therefore, there is no system for procuring these medications for these places.” (KI 1)  “Another barrier that we found is truly their lack of knowledge of how the delivery system works for delivering medicines to the health post. They don’t know how to. Therefore, when a piece of this chain is lost or is not working, then the whole distribution won’t work.” (KI 8) | Simplified drug tracking tools    Training and clearly defining roles for supply chain management    Decentralized system and telemedicine | “We learn how the supply chain works in each of those states and help the state governments in determining the estimating the medication they require both at the state, district, and at each facility level like giving them simple ready records…. Therefore, these are like simple ready reckoners pharmacists can refer to, based on their protocol and consumption patterns.” (KI 1)  “Therefore, for the first thing that we did was to understand the delivery system that they have, and then see what the bottlenecks in this system were and then try to fix them. In addition, then right now it’s working.... we needed to understand it and from [top] to bottom, make sure that everybody in the chain knows their role, and then is working.” (KI 8)  “What we’re working on is the decentralized scale, [which is working very well during the COVID time] to ensure these medications are available at [sub-centers] in India. [This] is a health facility where there are no physicians, but there are health workers who cater to approximately 5,000 population. These health workers can measure blood pressure and prescribe medication. [Patients] continue the medication if their blood pressure is under control, but if it is not under control, they’ll take the referral to the physician either physically ...but during COVID time, [we hope to continue referrals] through telemedicine.” (KI 1) |
| Inner Setting/Structural Characteristics | Quick turnover in leadership and staff | “Every state government has its own issues. Some issues—like changes in the leadership. [You] would go talk to a health secretary of a state and have all this conversation and convince [them to buy into your project]. [In] the next two days, you hear that the Secretary has changed. There’s a new person in there, then you have to start from zero.... In certain states, we had to wait for like 8 to 10 months before the things could be moved or [in some cases] the government collapsed in the state.” (KI 1)  “However, the other barrier is the personnel or staff turnover.” (KI 8) | Conducting ‘train the trainer’ sessions in ministries to help with onboarding new staff    Incentivizing staff | “Now we do ‘train the trainer’ inside the Ministry of Health System, [keeping track] of new people and mak[ing] sure [they get trained] ...because [we don’t], this [intervention] won’t work. There are [now] 235 new people who are part of this pilot program and have...receive[d] the right training. Therefore, the change is still work[ing].” (KI 8)  “Therefore, we paid them [field workers] independently of the government …at the same rate as what they would have got if the government was paying for those particular services. We [also] incentivize them with a per-meeting payment and they would get a bonus if more people came to the meeting.” (KI 3) |
| Outer Setting/  Patient Needs and Resources | Patient-level challenges of transportation and added lack of medications    Clients’ dislike for new food items    Low participant turnout, inconvenient timing, and loss of income due to (pre)intervention activities    Low coverage of intervention    Community reticence to engage with outsiders, as no trust has been built and champions were not present there, cultural and religious differences    Intervention unable to deal with related costs long-term    Mistrust due to prior negative research experience    Complicated intervention procedures    Subscribing to alternative medicine    Switching from public to private practitioners without monitoring    Rapid residential mobility among participants | “The first barrier was that the people expend to pay the transportation to go to the health facility….” (KI 7)  “Nepalese people are used to eating certain kinds of food and very attached to traditional food. Food is not just what we eat here; food is like a part of the culture, part of socialization, a part of festivals and everything and they thought that…. It would not be possible to change in foods.” (KI 2)  “Most likely, approximately 40% of people didn’t come to some of the group-based meetings. In addition, that would have been a timing issue.” (KI 3)  “I guess the biggest barrier was trying to engage with people to do the screening, mostly because people get a daily wage, they want to go out and work. They don’t get paid if they don’t go to work.” (KI 3)  “Therefore, people are very suspicious, some people, not all, but some are very suspicious of government agents. In addition, so, we were sometimes confused with government workers, and they wouldn’t want to tell us information like, you know, how many people live in a house and things like that…. Some of the villages too had different religions. You know, and some of them were more reticent than others, I think because they’re a minority group.” (KI 3)  **“**And then if you don’t know in Kano, there was a problem when Pfizer tried a drug [Trovan] in 1996. In addition, then there were some complications, it became a litigation issue. Yeah, so that truly affected us tremendously in Kano when we started research because if you mentioned research, [people] will think you’re bringing something new, to be tried on them as guinea pigs.” (KI 6)  “The complexity of what you’re doing in the research also affects, it could be a barrier to research.” (KI 6)  “Another challenge we have in intervention is alternative medicine. I mean [there are] drugs that claim [to have a] cure for everything, so we get patients [who participate in our research who subscribe to these alternative medicines].” (KI 6)  “The other problem is the big mobility [of] the people (participants) [due to seasonal] work. So [they] are moving from one city to another city [when it is] the time to collect coffee grain, [which] is one of the most important activities in Colombia.” (KI 7)  “Quite a percentage of the population would not return to the care. They go to the private practitioners, reasons of which we haven’t understood well.” (KI 1) | Convincing clients about the value-added to managing conditions rather than treating advanced conditions    Changing policy around medication prescription and use of polypill that is patient-friendly | “Therefore, one of the things that we taught the peers to do was to help patients think through, you know, tradeoffs, essentially to help them see that preventive measures may seem expensive now, it [won’t be] in the grand scheme of things, as it is actually going to be a cost-effective thing to do now. So that truly was what we came up with to try and address the issue of cost.” (KI 4)  “The medications for hypertension [were prescribed] for a very short duration of time, like seven days where the patients are expected to return after seven days to take their hypertension medication. Therefore, we had to change those policies to ensure at least 30 days of medications are prescribed and provided for free to these patients. Okay, so that was one of the patient-friendly things and so all the medications on the protocol are also the once-daily dosages so that it’s much easier for patients to take medication.” (KI 1) |
| Inner Setting/Culture | Existing cultural practices and socialization around food  Misconception about what healthy food means, and the asymptomatic nature of the disease, led to a reluctance to act | “Nepalese people are used to eating certain kinds of food and very attached to traditional food. Food is not just what we eat here; food is like a part of the culture, part of socialization, a part of festivals and everything and they thought that…it would not be possible to change in foods.” (KI 2)  “People thought that [they] already serve healthy food in the canteens. [They] serve clean, [they] wash the vegetables three times before [they] serve it. Their definition of healthy food was pretty different from our definition of healthy foods. In our initial conversation, they were reluctant because they all thought that [they] always serve healthy food, so [there was no need for the intervention].” (KI 2)  “The trouble with hypertension is that a lot of people don’t have symptoms. Therefore, even though they have hypertension, there’s a reluctance to do anything about it.” (KI 3) | Holding conversations to unify the meaning of healthy food | “Therefore, we had to do a couple of workshops with [stakeholders] to come to a common understanding of how we are defining healthy food and how they are defining it. Therefore, it took some time to come to a common ground.” (KI 2) |
| Inner Setting/Readiness for Implementation/Available Resources | Unavailability of new food items in the routine market    Loss of staff with specialized training in the private sector    Researchers performing multiple roles; lack of research space    Lack of research fund | “Brown rice was not available and for our intervention…. Similar to the brown rice, we went to like a prominent but small town where there were only three bakeries that were producing bread.” (KI 2)  “The other challenge I faced was where I trained some of my staff. In addition, then there are these private hospitals that spring up, and then my staff goes there. Therefore, I lost two members of staff that I trained particularly for CATH lab, they went to a private hospital.” (KI 9)  “For example, we don’t have adequate manpower for research. Now you are the investigator, you are the assistant, you are doing everything… We actually do most of the work ourselves. So that is a very big challenge.” (KI 6)  “The second barrier that we identify is the copayment of medication. Therefore, in Colombia, the health system gives some percentage of the cost of medication, but the people need to pay. It is one of the problems because when the project [finishes], the research money finishes…. They have no more, they need to go back to the standard health care.” (KI 7) | Meeting with local producers of healthy food items to arrange production and supply on the market | “Brown rice was not available and for our intervention, we had to talk to the local mills, and we asked them to produce brown rice for the hospital and the worksite. Similar to brown rice… there were only three bakeries that were producing bread. Therefore, we had a meeting with all of them, and... they were willing to make [the healthy bread] if somebody would buy it and we want to buy it, so they produced it.” (KI 2) |
| Non-CFIR Contextual Factor (Unanticipated Intractable Events) | COVID-19-related stalls and delays    Death of trained personnel | “The COVID-19 is a little disappointing because we were not able to reach out there because this first phase is supposed to be the household-related prevalence study where we enter the community to get details.” (KI 5)  “We trained sonographers from different parts of the country. However, unfortunately, one of the guys that I had trained died. Therefore, it was a big blow. Therefore, at the moment, I'm looking for a different person to train. The person who died was trained both in CATH lab work and ECHO. Therefore, you can imagine what a terrible loss.” (KI 9) |  |  |

**Supplement 4b.** CFIR and non-CFIR-mapped contextual facilitators of implementation feasibility in LMICs

| **CFIR Inner and Outer Setting Constructs**  **[Domain/Construct/Sub-construct]** | **Contextual Facilitators** | **Illuminating Statements from Key Informants** | **Reasons Facilitators Worked** | **Illuminating Statements from Key Informants** |  |
| --- | --- | --- | --- | --- | --- |
| Inner Setting/Readiness for Implementation/Leadership (Stakeholder) Engagement | Interest, buy-in, and support from leadership at multiple levels of government    Stakeholders’ buy-in and ownership    Overt national ownership and prioritization of intervention    Private stakeholders (pharma) support | “[We] talk[ed] to both people at the center and at the state and local involvement of the Indian Council of medical research, the then-director of the ICMR was first bought into this concept. Once that director agreed on what the message was, the concept was put across to the Secretary of Health in the country. Therefore, as I mentioned, the leadership, one was the buy-in by the leadership, which was the key important element [for us].” (KI 1)  “The most important thing was having contacts within the community who were excited and wanted to do something for the population that was outside what they normally do.” (KI 3)  “So being a national Task Force project, definitely, it’s a sign that you know, there is ownership for this project.” (KI 5)  “We have also some support from the pharmaceutical industry. They sell to us the medication because as the results are so good, and we have used the drugs that they expend, they have also contributed to the dissemination of the result of HOPE-4 between the community.” (KI 7) | Pharmaceutical companies provided meds because intervention outcomes have been so good; public and private stakeholders desire to see scale-up    Utilizing training and engagement approaches that empower implementers and stakeholders (community advisory boards)    Chain of authority is respected in these settings so buy-in from the top eases buy-in down the line | “We have also some support from the pharmaceutical industry. They sell to us the medication because as the results are so good, and we have used the drugs that they expend, they have also contributed to the dissemination of the result of HOPE-4 between the community.” (KI 7)  “I think engaging them [stakeholders] before we even had a fully refined intervention, engaging them and telling them…what we think would solve the problem, [wanting] to make sure we understand the problem from [their] perspective. Then, it is not like a token thing, because the Human-Centered Design Process [means] we [are working] based on what [stakeholders] tell us the problem is. We will also invite [them] to help us make sure we have an intervention that works.” (KI 4)    “We have a truly big collaboration of the people; when the people feel that there is some benefit and when people feel that it’s a serious work, without politician interest, the people collaborate very well.” (KI 7)  “First is the... you say the chain of authority...to follow the chain of authority is quite important because our system is not like the health director in a Health District takes the decision. No, they receive orders from above. Therefore, we have to go to the highest level and go down until we get to the health post. In addition, everything is—at least in Guatemala—everything should be written, and they have to receive an official letter who gets to get support to us when we get to the Health District, to the health center, to the health post.” (KI 8) |  |
| Inner Setting/Implementation Climate/Goals and Feedback; Learning Climate | Collaborators’ and researchers’ openness to learning during the implementation process and flexibility to change based on new knowledge | “I think our ability to learn from the program and change gears whenever required and that openness within the project implementers across the partnership was an added key [benefit].” (KI 1) | --- |  |  |
| Inner Setting/Implementation Climate/Compatibility | Leveraging the existing network of personnel and gatekeepers to implement and monitor interventions | “It was easier because we have enough resources to contact people who work in these places, particularly the non-physician healthcare workers that were people who live in the same counties where we implemented the protocol.” (KI 7)  “And, of course, the fact that the non-physician health workers go to homes each month to look if all is okay and take the blood pressure was another important thing.” (KI 7) | Communities were trusting of these personnel networks | “The second one was the participation of the non-physician healthcare workers that also increase the confidence of the community.” (KI 7) |  |
| Inner Setting/Readiness for Implementation/Access to Information and Knowledge | Efficient research staff monitoring intervention | “Another major factor was our research staff. They were on it! They were like—[they] were monitoring every week. We had a feedback session with the chefs and intervention group every month. Therefore, I think it was also continuously monitored. Therefore, I think that was also one of the major issues, major factors.” (KI 2) |  |  |  |
| Inner Setting/Implementation Climate/Relative Priority | Identifying and working with champions who believe in the intervention | “In the Rishi Valley, there are a couple of clinicians who I’ve been working with for many years, and they’re just dynamos and so it’s that contact, and having someone who’s excited and wants to do it is probably the most important thing. In addition, you know, and has the knowledge and interest and drive. Therefore, I think that’s probably the most important thing here.” (KI 3) | --- |  |  |
| Outer Setting/Patient Needs and Resources | Screening and diagnosing populations for conditions, who can then benefit from intervention | “I think ultimately, the thing that helps us get in there is the fact that the clinicians and the patients do see that there is a problem which does need to be addressed and they do believe that the intervention we designed can be able to address that ultimately comes down to those three things.” (KI 4) | --- |  |  |
| Outer Setting/Patient Needs and Resources    Inner Setting/Implementation Climate/Goals and Feedback; Learning Climate    Inner Setting/Readiness for Implementation/Leadership (Stakeholder) Engagement | Adopting a human-centered design approach    Early engagement    Regular interactions and feedback with implementers | “Because [of] the Human-Centered Design Process, what we are doing is based on what you tell us the problem, we will also invite you to help us make sure we have an intervention that works. Therefore, I think that also helped the Human-Centered Design Process helped because it included them.” (KI 4)  “I would say that regular interaction with them [implementers] and keep updated about the data being collected. In addition, that’s what...from past experience, I would suggest this. Regular interaction with all the level workers and staff and so that things are moving as we are interested.” (KI 5) | --- |  |  |
| Outer Setting/Cosmopolitanism    Outer Setting/External Policies and Incentives | Existing successful collaborations and presence with communities and legitimate ministries and agencies | “You have to know the critical stakeholders to do that. When you convince the right people, they do the rest. In addition, they know better than you how to do it.” (KI 8)  “And because our vice minister was like the first to believe in our project, he supported us at that time.” (KI 8) | Communities were already confident because of prior positive interactions with the research group, trust, and confidence in the researchers | “I am a very well reputed medical doctor, and my institution has a good reputation also, which improved this facility to gain the confidence of the population.” (KI 7) |  |
| Inner Setting/Implementation Climate/Relative Priority; Tension for Change | Collective acknowledgment of need and the intervention’s ability to address the need | “We have a truly big collaboration of the people; when the people feel that there is some benefit and when people feel that it’s serious work, without [political] interest, the people collaborate very well. We thought we got truly good, a very good collaboration of the people.” (KI 7) |  |  |  |
| Inner Setting/Readiness for Implementation/Available Resources | Availability of funding    Sufficient resources for designated sites | “Well, with HOPE-4, we had good financial support for the implementation of the study, it was easier than in the PURE study.” (KI 7)  “This is because we have multiple funding agencies in India, we’ve applied for one of the funding agencies, and we got the grant from the agency.” (KI 5)  “Additionally, because it was developed only in one site, only in one department, only in one city. Therefore, we use different counties from the same city, so it is easier for the follow-up of the patient. It was easier because we have enough resources to contact people who work in these, particularly the nonphysician healthcare workers that were people who live in the same counties where they implemented the protocol.” (KI 7) | --- |  |  |
| Outer Setting/External Policies and Incentives | Changing national treatment guideline recommendations that align with intervention components (e.g., polypill) | “More of the guidelines now are recommending the use of a fixed-dose combination of antihypertensive drugs, that for us was good because you lower the risk of secondary effects when we use two drugs combined in the same tablet. Now more of the guidelines have recommended the use of this, it is easier to improve the adherence and decrease the secondary effect.” (KI 7) | Because changing guidelines improves client experience by reducing secondary effects of multiple pills and increasing medication adherence | “Now more of the guidelines have recommended the use of this,  it is easier to improve the adherence and decrease the secondary effect.” (KI 7) |  |
| Inner Setting/Readiness for Implementation/Relative Priority; Tension for Change | Visible demand for intervention    Rarity of disease conditions on a global scale; a rare opportunity for knowledge creation through research    Improved research capacity and advocacy at the national level    Clearly, defined health professional agenda for CVD research    Acceptance of intervention and results by professional societies | “I think ultimately, the thing that helps us get in there is the fact that the clinicians and the patients do see that there is a problem which does need to be addressed and they do believe that the intervention we designed can be able to address that ultimately comes down to those three things.” (KI 4)  “The fact is that our research is answering a question that nobody knows the answer to globally. Therefore, it’s easy to sell… Rheumatic heart disease is very common in Africa. Is it more than in the West? … Actually, this research cannot even be done in the West because there are no patients with this [disease].” (KI 6)  “I could say that many of us cardiologists in Nigeria have worked very hard to improve the research capacity of people in the country.” (KI 6)  “And also, we have come together and defined an agenda for us in the country as a group. In addition, we are now trying to answer a question that we didn’t have answers to before in our population. Therefore, this has influenced policy and, we are now even going a step forward to engage the policymakers; we’re doing advocacy with the Ministry of Health in Nigeria, to show them what we were doing and how they can also participate.” (KI 6)  “The second one is the medical academic societies accept well the result of the HOPE 4 and was not creating controversy…to the use nonmedical workers in the control of hypertension” (KI 7) | --- |  |  |
| Outer Setting/Patient Needs and Resources | Providing patient-friendly incentives such as access to additional and free tests and meds and home visits | “The people receive some activities free. For instance, the care of the blood pressure taking in the house and the free medication and the recommendation and it is important also.” (KI 7)  The fact that you’re able to give these patients this drug-free, both the warfarin and rivaroxaban are given free, so at least they’re getting some medications free, they are getting investigations done free, [especially] in the context of a system whereby you pay out of pocket.” (KI 6) | Incentivized follow-up and participants showing up for intervention sessions | “Therefore, I think that was partly a facilitator in that they got something back. In addition, one of the sites, we also did some finger prick blood tests as well like lipids and glucose, which was a real incentive for them to come because normally that would cost them money and that didn’t cost them money. So that was a facilitator.” (KI 3) |  |
