# Supplementary material for: The CATALYTIC tool to assess feasibility of implementing evidence-based interventions for cardiovascular diseases in 46 low- and middle-income countries: survey outcomes and tool reliability testing
Source: Front Public Health. 2025 Dec 10;13:1597996. doi: 10.3389/fpubh.2025.1597996 (PMC12727921; doi:10.3389/fpubh.2025.1597996)
Supplement: Supplementary file 5 [file Table_5.docx]

**Supplement 5.** Survey items and their corresponding CFIR or inductive code

| **Inner Setting Constructs (with definitions)** | **Survey Items** | |
| --- | --- | --- |
| Structural characteristics (social architecture, age, maturity, size of an organization) | What is the extent of influence of ____ on feasibility?  1. The availability of physical infrastructure.  2. Flexible administrative operations at the facility or organization that houses the intervention.  3. Being able to leverage existing infrastructure at intervention site(s).  4. Being able to leverage the existing workforce at the intervention site(s) to deliver the intervention. | |
| Network and Communications (nature and quality of networks and communication) | Questions that rated the quality of contextual factors…  1. How would you rate the quality of communication between those who implement your intervention and the organizational leadership?  2. How would you rate the quality of communication between those who implement your intervention and those who participate in it?  3. How would you rate the quality of interaction between those who implement your intervention and the organizational leadership?  4. How would you rate the quality of interaction between those who implement your intervention and those who participate in it? | |
| Culture (norms, values, and basic assumptions of an organization) | What is the extent of influence of …?  1. Presence of supportive interpersonal relationships (for example relationships with family members, friends, or co-workers).  2. Presence of supportive community culture.  3. Promoting an empowering narrative around the intervention  4. Promoting an empowering environment around the intervention.  5. Being well-known and recognized by the intervention’s target population and stakeholders.  6. My institution/organization is well known and recognized by the intervention’s target population and stakeholders. | |
| Implementation Climate (absorptive capacity for change)  ---Tension for Change  ---Compatibility  ---Relative Priority  ---Organizational Incentives and Rewards  ---Goals and Feedback  ---Learning Climate | What is the extent of influence of _____ on the feasibility?  1. Intervention’s implementation process being conducive to implementers’ routine workflow.  2. Intervention’s implementation process being conducive to participants’ daily routine.  3. Other healthcare professionals’ recommendations for the intervention.  4. Other healthcare professionals’ confidence in and support of the intervention.  5. Being able to leverage community members to implement the intervention.  6. Healthcare professionals’ perceptions of the intervention’s usefulness.  7. Setting a minimum requirement of expectations for those implementing the intervention.  8. Setting a minimum requirement of expectations for participants during the intervention.  9. Poor remuneration of healthcare personnel involved in the intervention.  10. Easy channels for providing feedback on the intervention.  11. Information gotten from implementers’ feedback on the intervention.  12. Information gotten from participants’ feedback on the intervention.  13. Inclusive learning strategies (e.g., training, certification programs, workshops, consultations) available to implementers.  14. The training style for preparing implementers.    Questions that rated the quality of contextual factors…  15. How would you rate the convenience of the intervention’s implementation process to implementers’ routine workflow?  16. How would you rate the implementers’ experience of giving feedback about the intervention?  17. How would you rate the participants’ experience of giving feedback about the intervention?  18. In your experience, which of these training styles are most effective when preparing implementers for your intervention?  (a) Didactic (primarily instructional training that may include an instruction manual)  (b) Iterative (training that allows repetition of steps to further adjust, improve, and master content and processes)  (c) Experiential (emphasis on trainees learning from experience by doing practice runs of actual tasks that are as close to the real-life scenario of the implementation process)  (d) Empowering (training that encourages trainees to take initiative on, contribute to, and modify training content and processes as a way to make training more effective and build buy-in among trainees) | |
| Readiness for Implementation (organizational commitment to implement)  ---Leadership Engagement  ---Available Resources  ---Access to Knowledge and Information | What is the extent of influence of ____ on feasibility…?  1. Leaders’ and key stakeholders’ engagement and support for intervention  2. Unsuccessful stakeholder engagement  3. Provision of training to implementers  4. Provision of equipment for implementing intervention  5. Use of technology and other tools for implementers’ access to information and knowledge about the intervention and the implementation process  6. Information regarding the intervention and its implementation is comprehensible to implementers  7. Information regarding the intervention and its implementation is comprehensible to participants  8. Information and knowledge regarding the intervention and its implementation being accessible to implementers  9. Information and knowledge regarding the intervention and its implementation being accessible to participants  Questions that rated the quality of contextual factors…  10. In your opinion, how involved should leaders and key stakeholders be with implementing an intervention in an LMIC setting?  11. In your experience, how much effort do you put into negotiating with and bringing critical yet reluctant stakeholders to support your intervention?  12. In your opinion, when is it a good time to engage stakeholders in the implementation process?  13. In your experience, which of these styles of stakeholder engagement worked best for you? Response options are:  (a) Top-down relationship building (getting in touch with most senior personnel in ministries, communities, institutions, and organizations)  (b) Peer-to-peer relationship building  (c) ‘Bottom-up’ grassroots relationship building  14. In your experience, which types of relationships influence stakeholder engagement? Response options are: ‘Personal,’ ‘Professional,’ ‘A combination of both personal and professional’  15. How would you rate the implementers’ access to information and knowledge to optimally implement the intervention?  16. How many resources were available at your institution for implementing the intervention?  17. How many resources did you have to provide to implement your intervention?  18. In your experience, which of these resource scenarios are you most familiar with in terms of implementing interventions? Survey responses are Steady, Periodic, Grant-by-grant basis, Trickle, and Rare. | |
| **Outer Setting Domain (with definitions)** | **Survey Items** | |
| Patient Needs and Resources (barriers and facilitators to meeting needs) | 1. In your opinion, being unable to address participants’ needs within the bounds of your intervention is a deal-breaker for implementing it? | |
| Cosmopolitanism (degree of organizational networking) | What is the extent of influence of ____ on feasibility…?  1. Collaborating with community organizations for the intervention | |
| Peer Pressure (competitive pressure to implement) | What is the extent of influence of ____ on feasibility…?  1. Other ongoing health activities similar to the intervention in the same locale | |
| External Policies and Incentives (external strategies to implementation) | What is the extent of influence of ____ on feasibility…?  1. Existing government policies/ regulations/guidelines/politics  Questions that rate the quality of contextual factors…  2. How supportive are existing government policies towards implementing your intervention in this setting?  3. How disruptive are existing policies to implementing your intervention in this setting?  4. How likely will findings from your study to reshape or improve the existing health policies in this setting? | |
| The two inductive codes are as follows: | | |
| **Inductive Codes (with definitions and thematic summary)** | | **Survey Items** |
| Context driving intervention needs and design  Pre-conceived awareness of contextual challenges to implementation and acceptability of interventions, pre-implementation.    *An observation during interviews was that key informants had an early awareness of contextual barriers to the successful implementation of their interventions and reception of the interventions by their intended beneficiaries. As such, key informants, before piloting/implementing their interventions, conducted baseline assessments that helped them identify these specific barriers, which in turn inspired/informed the design of their intervention and implementation process. | | What is the extent of influence of ____ on feasibility…?  1. Conducting a needs assessment or contextual evaluation of our intervention setting before implementation  2. Before implementing an intervention, it is acceptable for the formative evaluation to be time-consuming  3. Identifying barriers before implementing my intervention is informative to me.  4. Identifying barriers before implementing the intervention informs the strategies, we use to deliver the intervention.  5. Identifying barriers while implementing the intervention is informative to me.  6. Being able to identify implementation barriers before implementation (pre-implementation).  7. Being able to identify implementation barriers while delivering the intervention (during implementation).  8. I am flexible with my implementation protocol (i.e., being open to making changes) at the pre-, during, and post-implementation stages of the intervention. |
| Unplanned events in the implementation process  Unexpected and intractable events that occur  *Given that the interviews were conducted at the height of the COVID-19 pandemic in 2020, key informants reported an abrupt stop to research activities, sometimes at the cusp of advancing innovative tools during the intervention. Other unforeseen circumstances were the loss of life of research personnel, who had received highly specialized training as part of the intervention and were difficult to replace. | | What is the extent of influence of ____ on feasibility…?  1. 1. Unforeseen events (for example COVID-19 pandemic, sudden loss of life, loss of funding, natural disasters, and change in national leadership) being disruptive to my intervention. |
