# Supplementary material for: The CATALYTIC tool to assess feasibility of implementing evidence-based interventions for cardiovascular diseases in 46 low- and middle-income countries: survey outcomes and tool reliability testing
Source: Front Public Health. 2025 Dec 10;13:1597996. doi: 10.3389/fpubh.2025.1597996 (PMC12727921; doi:10.3389/fpubh.2025.1597996)
Supplement: Supplementary file 6 [file Table_6.docx]

**Supplement 6.** Number of rotated factors, with corresponding number of items, identified from EFA

| **Factor Number** | **Number of Items with Factor Loading >0.3** |
| --- | --- |
| Factor 1 | 3 |
| Factor 2 | 2 |
| Factor 3 | 2 |
